# Diagnostics of lung cancer by fragmentated blood circulating cell-free DNA based on machine learning methods

**DOI:** 10.3389/fmed.2025.1435428

**Published:** 2025-01-29

**Authors:** Ivan O. Meshkov, Alexander P. Koturgin, Pavel V. Ershov, Liubov A. Safonova, Julia A. Remizova, Valentina V. Maksyutina, Ekaterina D. Maralova, Vasilisa A. Astafieva, Alexey A. Ivashechkin, Boris D. Ignatiev, Antonida V. Makhotenko, Ekaterina A. Snigir, Valentin V. Makarov, Vladimir S. Yudin, Anton A. Keskinov, Sergey M. Yudin, Anna S. Makarova, Veronika I. Skvortsova

**Affiliations:** ^1^Federal State Budgetary Institution “Centre for Strategic Planning and Management of Biomedical Health Risks” of the Federal Medical and Biological Agency (Centre for Strategic Planning, of the Federal Medical and Biological Agency), Moscow, Russia; ^2^The Federal Medical and Biological Agency (FMBA of Russia), Moscow, Russia

**Keywords:** machine learning methods, lung cancer, fragmentome, circulating cell-free DNA, cfDNA, diagnostic classification model, cancer early detection

## Abstract

**Introduction:**

Minimally invasive diagnostics based on liquid biopsy makes it possible early detection of lung cancer (LC). The blood plasma circulating cell-free DNA (cfDNA) fragments reflect the genome and chromatin status and are considered as integral cancer biomarkers and the biological entities for ‘cancer-of-origin’ prediction. The aim of this work is to create a method for processing next-generation sequencing (NGS) data and an interpretable binary classification model (CM), which analyzed cfDNA fragmentation features for distinguishing healthy subjects and subjects with LC.

**Methods:**

148 healthy subjects and 138 subjects with LC were included in the study. cfDNA fractions, isolated from blood plasma biospecimens, were used for DNA libraries preparations and NGS on the NovaSeq 6,000 Illumina system with a coverage of 100 million reads/sample. Twelve variables, describing the abundance and length distribution of cfDNA fragments within each genomic interval, and 40 variables based on the values of position-weight matrices, describing combinations of 5-bp-long terminal motifs of cfDNA fragments, were used to characterize genomic fragmentation. Classification models of the first phase of machine learning were based either on logistic regression with L1- and L2-regularization or were probabilistic CMs based on Gaussian processes. The second phase CM was based on kernel logistic regression.

**Results:**

The final CM can distinguish healthy subjects and subjects with LC with AUC values of 0.872–0.875. The performance of developed CM was evaluated using datum and testing sets for each LC stage category. Sensitivity values ranged from 66.7 to 85.7%, from 77.8 to 100%, and from 70 to 80% for LC stages I, II, and III, respectively. Specificity values ranged from 79.3 to 90.0%.

**Discussion:**

Thus, the CM has a good diagnostic value and does not require clinical or other data on tumor-associated biomarkers. The current method for LC detection has some advantages for future clinical implementation as a decision-making support system due to the performance of the CM requires data exclusively from NGS-analysis of blood plasma cfDNA fragmentation; the accuracy of the CM does not depend on any additional clinical data; the CM is highly interpretable and traceable; CM has appropriate modular architecture.

## 1 Introduction

Lung cancer (LC) is one the major public health challenges in many parts of the world. According to GLOBOCAN (1), the number of new cases of LC and the number of LC’ deaths were about 2.2 and 1.8 million, respectively, in 2020. Luo and colleagues (2) forecast that the number of annually diagnosed LC cases in 40 countries of the world will grow by about 65% (from 1.31 million in 2010 to 2.17 million) in 2035. A more distant global forecast for 2050 is even less optimistic and indicates that LC incidence and mortality will achieve 3.8 and 3.2 million cases, respectively (3). Tobacco smoking still remains the most significant risk factor for LC (4). Therefore, in-time and early detection of the LC in a wide population exposed to a complex of external factors of high carcinogenic risk is a very reasonable cancer preventive strategy that will reduce public health burden.

Regular LC screening is implemented through national medical programs based on the radiation diagnostics, which has its advantages and disadvantages (5). The latter are associated mainly with the risk of negative consequences of radiation exposure that described in reviews (6, 7). Recently, great scientific progress has been made in the molecular profiling of circulating cell-free DNA (cfDNA) in peripheral blood. cfDNA analysis is being considered as a safer diagnostic alternative to low-dose computed tomography of the lung or additional option in the clinical decision-making support system (8). It is of fundamental importance that presence of cfDNA in blood is an integral indicator reflecting the occurrence of many physiological (9) and pathological processes in the body (10), in particular, LC (11).

A pool of blood cfDNA is represented as a set of DNA fragments of different lengths, which are resulted from the processes of cell death. The appearance of cfDNA fragments is also associated with a rather complex biology and context-specific regulation of gene expression (12, 13). Thus, the cfDNA fragments (fragmentome) reflects the current state of the genome and chromatin. We mean a term ‘fragmentome’ as the entire set of cfDNA fragments present in a particular sample, although there is no well-established interpretation of this term yet (13). Apoptosis-associated cfDNA is usually represented by fragments of 166 and 320 bp in length in circulating blood (14), which corresponds to mono and dinucleosomal clusters, respectively. However, in reality, blood cfDNA fragments are distributed in a wider range (from 40–200 bp to 180–1,000 bp) (15).

We have summarized below well-known parameters of cfDNA. Avanzini and colleagues calculated that, on average, 0.014% of DNA enters the bloodstream as a result of a single tumor cell death (16). A fraction of circulating tumor DNA (ctDNA) directly releasing from tumor cells can be estimated. The total amount of ctDNA might be <0.01% of the total cfDNA concentration (17) but the later tumor stage the more increasing ctDNA percentage. According to various studies, the fraction of ctDNA constitutes ~0.1–89% of cfDNA (15). ctDNA fragments are represented as a combination of short and long fragments, which ratio, obviously, can vary depending on tumor localization and a leading cellular process that contributes to fragmentation of genomic DNA. Thus, ctDNA turned out to be a more fragmented fraction compared to non-tumor cfDNA fragments, e.g., <100 bp in length (18). Similar results were found in (19) where isolated cfDNA from patients with colorectal cancer was enriched in short fragments of 90–150 bp in length (sub-mono-nucleosome cluster).

Despite the clear clinical significance of ctDNA analysis in peripheral blood or other body fluids, there is still no consensus on the advantages of ctDNA detection over imaging methods of cancer detection. Pons-Belda and colleagues (20) conclude that current methods of ctDNA detection have the potential to predict a tumor foci larger than 10–15 mm in Ø, which is comparable to the sensitivity of imaging methods. In another work, the performance of mathematical model based on ctDNA content for prediction of small tumor foci (0.83 cm) was demonstrated (16). A high predictive value of ctDNA has been also shown. Tumor treatment failure can be predicted 140 days earlier than it is detected by imaging methods (16).

Since ctDNA in the early tumor stages is present in extremely low concentrations [e.g., genetic alterations in tumors can be detected when the ctDNA content is >5% of total cfDNA (14)] and the blood ctDNA has a short lifetime (minute-hour interval) (15), it is expedient to use information about the fragmentation pattern of total cfDNA. The term ‘fragmentation pattern’ is meant a system of characteristics of target cfDNA fragments (fragment lengths, ratio of different fragments lengths, and nucleotide sequences of fragments). This will help to overcome the aforementioned diagnostic limitations of ctDNA in the early tumor stages and ensure proper sensitivity and specificity of mathematical classifiers for separation of normal and tumor cases in accordance with the fragmentation patterns of total cfDNA. Data on the fragmentation patterns of cfDNA from body fluids are required in the framework of minimally invasive cancer diagnostics and to increase interpretability of small tumor foci to exclude false positive or false negative results of radiation diagnostics (e.g., a low-dose computed tomography data).

Formally, a new era in the analysis of cfDNA fragmentome can be associated with works by Cristiano and colleagues (21) and Mathios and colleagues (22). They reported usage of a universal DELFI algorithm (DNA EvaLuation of Fragments for early Interception) based on machine learning methods to process NGS-datasets. It performs through the mapping of fragment sequences to the genome in 5 million bp non-overlapping regions, and within each region it is possible to study the coverage and distribution of short (100–150 bp) and long (150–220 bp) cfDNA fragments in groups of healthy controls and subjects with cancers (21). Later, the validity of the DELFI algorithm in combination with several additional clinical parameters was demonstrated to diagnose LC stages I/II and III/IV, respectively, at 91 and 96% sensitivity (80% specificity) (22).

There is an approach, which operates cfDNA fragments distribution and nucleotide sequences of end motifs of cfDNA fragments. Its applicability is described by Guo and colleagues (23). They generated a motif-based breakpoint model at the 5′ end of each cfDNA fragment to distinguish between healthy subjects and subjects with LC at early stage using three different methods (logistic regression with elastic network regularization, deep learning, and extreme gradient boosting) (23). Later, the model was improved by including multiple cfDNA fragmentome characteristics such as gene copy number, cfDNA fragment coverage (FSC) and size distribution (FSD), terminal motif sequence data (EDM), and breakpoint motifs (BPM) (24). The addition of multiparametric characteristics made it possible to increase the sensitivity of the model in detecting lung cancer (stage I) and tumor foci <1 cm to 83.2 and 85.0%, respectively (24). The same group proposed the model not only to distinguish healthy controls from subjects with cancers (AUC ≈ 0.98), but also adapted the model to determine the tumor localization by cfDNA having tested it on three different cancer types (an overall accuracy = 93%) (25).

Thus, there is strong evidence that classification models based on the fragmentation pattern of cfDNA have high diagnostic value in the detection of LC. It follows from the existing literature data that at least classification models using multiple characteristics, in addition to fragmentation patterns of cfDNA, have better sensitivity and AUC values. At the same time, the need to collect a large amount of medical information about clinical characteristics, risk factors, and cancer biomarkers will make it very difficult to apply complex classification models in a clinical practice and lengthen the waiting time to get the analysis result. As far as we know, no entirely interpretable machine learning classification models have been described that allow to separate different groups of subjects with high accuracy and excellent quality operating with NGS-data on cfDNA fragmentation pattern only. Development of such a model, based on a combination of machine learning methods, will overcome some limitations in low ctDNA concentrations in early LC detection, as well as overcome usage of information about multiple biomarkers and clinical patients’ data in addition to cfDNA fragmentome data. Therefore, the aim of this work is to create a universal method for processing NGS-data and to design an interpretable binary classification model for distinguishing between healthy subjects and subjects with LC according to fragmentation pattern of cfDNA isolated from the blood plasma.

## 2 Materials and methods

### 2.1 Study design and clinical data

The study involved subjects who met the inclusion criteria. Subjects were divided into two equal cohorts: healthy subjects (control cohort) and subjects with confirmed LC (cancer cohort). The ratio of men and women in the cohorts was about the same.

The inclusion criteria in the control cohort were as follows: signed informed consent approved by the ethics committee; men and women aged from 40 to 77 years, inclusive; non-smokers and never smoked Caucasians; no history of oncological, hematological and autoimmune diseases; no exposure to genotoxic environmental factors (silica dust, radon, cadmium, asbestos, arsenic, beryllium, chromium, nickel, coal smoke, soot) and occupational hazards associated with prolonged exposure to vehicle exhaust; absence of acute and chronic diseases of the respiratory system; absence of active pulmonary tuberculosis; absence of hepatitis B, hepatitis C, HIV; absence of systemic therapy, with the exception of nonsteroidal anti-inflammatory drugs (NSAIDs), antiplatelet agents and anticoagulants, as well as drugs for the treatment of coronary heart disease and hypertension.

The inclusion criteria in the cancer cohort were as follows: signed informed consent approved by the ethics committee; men and women >18 years old; the presence of clinical signs and indications for surgery or biopsy for primary LC, obtained on the basis of computed tomography or other methods of radiation diagnostics; histological confirmation of the diagnosis after biopsy/resection with an assessment according to TNM classification; the presence of measurable tumor foci; satisfactory liver function (bilirubin <2× upper normal limit); increase in ALT (SGPT) and AST (SGOT) to no more than 2.5× upper normal limit (no more than 5× upper normal limit in patients with metastatic liver damage); satisfactory kidney function (creatinine ≤1.5× upper normal limit and/or creatinine clearance according to the Cocraft-Gault formula >50 mL/min); adequate bone marrow function expressed in the following peripheral blood parameters: neutrophil count >1,500 ×106/l; platelet count >75 × 106/l; hemoglobin content >90 g/L; absence of treatment for lung cancer in the past; absence of another oncological disease in the anamnesis over the past 5 years; absence of clinical signs indicating the secondary nature of tumor formations; absence of severe condition of the subject; absence of active pulmonary tuberculosis; absence of hepatitis B, hepatitis C, HIV; absence of pregnancy on at the time of the study and during the last 5 years (for women); absence of hematological pathologies.

### 2.2 Collection of biospecimens

Collection of biospecimens and clinical data from the subjects recruited for this study was approved by the independent local ethics committee at Arte Med Assistance LLC: Protocols no. 253 of May 26, 2021 and no. 276 of May 11, 2022, and performed in accordance with the Helsinki Declaration (Protocol no. 05112019 of 11.11.2019). All subjects gave written informed consent after a full explanation of all procedures.

Peripheral venous whole blood samples from the subjects included in the study were collected in 10 mL PAXgene Blood ccfDNA tubes (QIAGEN, Germany). The tubes contain additives preventing blood clotting and stabilizing blood cells for excluding contamination by intracellular genomic DNA during storage. Thirty milliliters of blood were collected from each subject. After that, the tubes were immediately mixed manually with smooth movements, without shaking the tubes in accordance with the manufacturer’ guidelines. Storage of samples was carried out at +4°C to 10°C within 7 days.

### 2.3 Isolation of blood plasma fraction

The blood plasma fraction was obtained according to the PAXgene Blood ccfDNA Tubes (QIAGEN, Germany) manufacturer’s protocol. The PAXgene Blood ccfDNA Tubes were centrifuged for 15 min at room temperature (15–25°C) and 1900 × g. The supernatant (plasma) was transferred to 15 mL conical bottom centrifugation tubes and centrifuged at room temperature for 10 min at 1900 × g. The supernatant (plasma) was pipetted into cryo tubes without disturbing the residual blood cell pellet at the bottom of the tube, if present. Cryo tubes were sent for long-term storage at −80°C.

### 2.4 The isolation of circulating cell-free DNA

Isolation of circulating cell-free DNA (cfDNA) from blood plasma samples was performed using QIAamp Circulating Nucleic Acid Kit (QIAGEN, Germany) according to the manufacturer’s protocol. The isolated cfDNA was stored at −20 °С temperature. cfDNA concentration was measured using the Quantus fluorometer (Promega, United States) with QuantiFluor ONE dsDNA fluorescent dye (Promega, United States). The purity of the isolated cfDNA was assessed using the NanoDrop 8,000 spectrophotometer (Thermo Fisher Scientific, United States) by measuring absorbance on different wavelengths and calculating the A260/A280 and A260/A230 ratios. Fragment length distribution of cfDNA was assessed by capillary electrophoresis using the Agilent 4,200 TapeStation system (Agilent Technologies, United States).

### 2.5 NGS analysis of the fragments of cfDNA

DNA libraries for NGS analysis were prepared with the NEBNext Ultra II DNA Library Prep Kit for Illumina (New England Biolabs, United States). The concentration of the libraries was measured using the Qubit fluorometer (Thermo Fisher Scientific, United States) with the Qubit dsDNA 1X HS Assay Kit fluorescent dye. The length of the libraries was determined by an Agilent TapeStation 4,200 system using the D1000 ScreenTape Assay (Agilent Technologies, United States).

Sequencing was performed on the NovaSeq 6,000 Illumina system (Illumina, United States) with the NovaSeq S1 reagent kit (200 cycles) v1.5 and NovaSeq S2 reagent kit (200 cycles) v1.5. A PhiX adapter-ligated library was used as a control. Sequencing was carried out according to the manufacturer’s protocol with a coverage of at least 100 million reads per sample.

### 2.6 Bioinformatics processing of NGS-data

The first characteristic of genome fragmentation is the pattern of cfDNA fragments distribution by lengths and their ratio. To transform NGS-data on the blood plasma cfDNA fragmentome into characteristics of genome fragmentation, several stages of processing the output NGS-data were performed.

**Figure 1:**
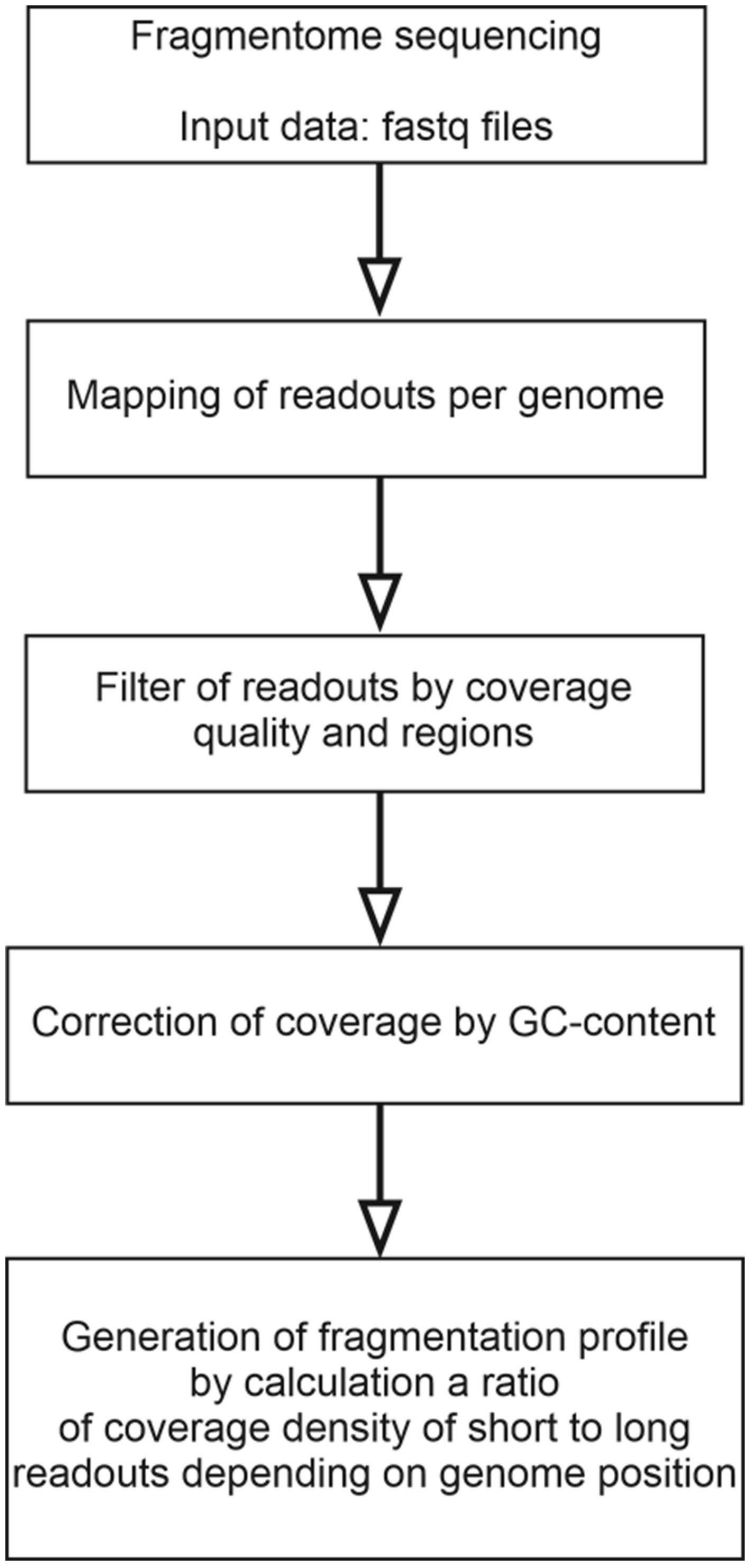
A flowchart of the algorithm for bioinformatics processing of NGS data on the cfDNA fragmentome.

cfDNA fragmentome reads were mapped to the human reference genome (hg38) and then filtered by mapping quality. Reads with a MAPQ ≤30 (MAPping Quality score), polymerase chain reaction (PCR) duplicates, and reads located in areas of ambiguous mapping [telomeres, centromeres, and those included in the ENCODE Black-list (26)] were removed. The genome was divided into 100,000 bp-intervals, within which the number of short (100–150 bp) and long (150–220 bp) fragments was calculated. The number of fragments obtained were adjusted for guanine and cytosine content (GC-content) according to the protocol described by Benjamini and colleagues (27). This method for constructing a fragmentation pattern differs from the DELFI method by Cristiano and colleagues (21). The final result of DELFI is a set of ratios of the number of short to long fragments that undergo double correction for GC-content: first, the correction is applied to the number of short and long fragments, then to the resulting ratio of the corrected number of fragments. In the current study, correction is applied only once, and its result is the corrected numbers of short and long fragments for each genomic interval under consideration. A schematic representation of the algorithm of bioinformatics processing of NGS-data is shown in Figure 1.

The second characteristic of genome fragmentation is a set of position-weight matrices (PWMs), which are calculated according to the method by Claverie and colleagues (28). In the current study, PWMs are used to characterize the terminal motifs of cfDNA fragments. The PWM contains information about motifs by which DNA fragmentation most often occurs within a single 100,000 bp genomic interval. The PWM is a matrix in which the number of rows corresponds to four nucleotides, and the number of columns corresponds to the length of the motif. From each 100,000 bp genomic interval, 5-bp-long terminal motifs of cfDNA fragments were extracted from 5`- and 3`-ends. From these motifs, an alignment was compiled for each genomic interval and then converted into a PWM. For one sample analyzed, a set of such PWMs was compiled for all genomic intervals.

### 2.7 Generation of training, datum and testing datasets

The total number of 286 subjects were included in the current study. To train and test the performance of the model, they were stratified by sex, age and the presence of LC (the cancer stage was also taken into account), and then this array was randomly divided, taking into account stratification, into three datasets in the ratio 60%: 20%: 20% for training, datum and testing datasets, respectively (Supplementary Figure S1A, Tables S1–S6). The training dataset included 60% of the total number of subjects and was used to train the model. The datum dataset included 20% of the total number of subjects and was used both to test the performance of the model and to generate new variables at cer-tain stages of constructing the classification model. The testing dataset included 20% of the total number of subjects and was used for the final verification of the model’s performance.

### 2.8 Description of the study variables

The target cfDNA fragmentome is a pool of DNA fragments from 100 to 220 bp mapped to the genome. In turn, the genome is divided into non-overlapping intervals and a certain number of DNA fragments are found within each of them. Many DNA fragments within one genomic interval can be characterized from two sides at once.

First, 12 variables were used to describe the abundance of DNA fragments and the distribution of their lengths within each genomic interval. The total number of DNA fragments, the number of short fragments (100–150 bp), the number of long fragments (151–220 bp) and the logarithm of the ratio of the number of short fragments to the number of long fragments were considered. Since a situation is possible when either long or short fragments are absent in a particular genomic interval, it was decided to limit the resulting logarithm value to limits from −10 to +10. Variables, characterizing the total number of fragments, the number of short and long fragments, taking into account changes in the GC-content throughout the genome, were considered. In addition, the characteristics of the distribution of fragment lengths were assessed for each genomic interval: mean, standard deviation, skewness (asymmetry), kurtosis (kurtosis coefficient, a numerical characterization of the degree of sharpness of the peak of the distribution of a random variable) and standard error of the mean.

Secondly, variables based on the values of PWM were used. The NGS-data is divided into 26,460 non-overlapping intervals. In turn, each interval is characterized by 40 variables that correspond to PWM elements (4 nucleotides*2 ends of the fragment*5 positions at each end). Using the principal component analysis (PCA), it is possible to perform dimensionality reduction and replace 40 variables with a smaller number, while losing as little information as possible. To reduce dimensionality, a datum dataset was used.

The dimensionality reduction procedure is presented in Supplementary Figures S1B, S2. Briefly, the datum dataset was initially considered a table consisting of n rows corresponding to observations and 26460*40 columns corresponding to variables. The datum dataset was transformed into a table, where a separate region of a subject became an observation; the columns began to correspond to the elements of PWM. The resulting table had a dimension of 26460*n rows by 40 columns and was converted using PCA. Using a scree plot, m informative principal components were identified (m < 40). The calculated principal components are linear combinations of elements of PWM and will be referred to hereinafter as “fragmentation pattern.” The formula used to calculate an individual fragmentation pattern is the same for all genomic intervals and for all subjects, so the fragmentation pattern reflects the characteristics of the terminal motifs of cfDNA fragments on a metagenomic scale. As a result, for each subject and for each genomic interval it is possible to replace 40 variables, corresponding to PWM elements, with m variables, corresponding to the fragmentation patterns (m < 40).

The formulas, for calculating fragmentation patterns, were obtained solely on the basis of observations from the datum dataset; no information from the training or testing datasets was used in their calculation.

### 2.9 Preliminary description of the classification model architecture

Metamodeling is used to generate a classification model in the current study. This method is widely used in machine learning and adapted for generation of several dozens of classification models at the first phase that are relatively easy to interpret. Each model separately may demonstrate mediocre results for classification of observations. In addition, the model generated at the first phase of machine learning may not use the entire set of features but uses a part of them, for example, the distribution of cfDNA fragments lengths or fragmentation pattern. In the second phase, the results of the models generated at the first phase are used as variables to build the final model.

**Figure 2:**
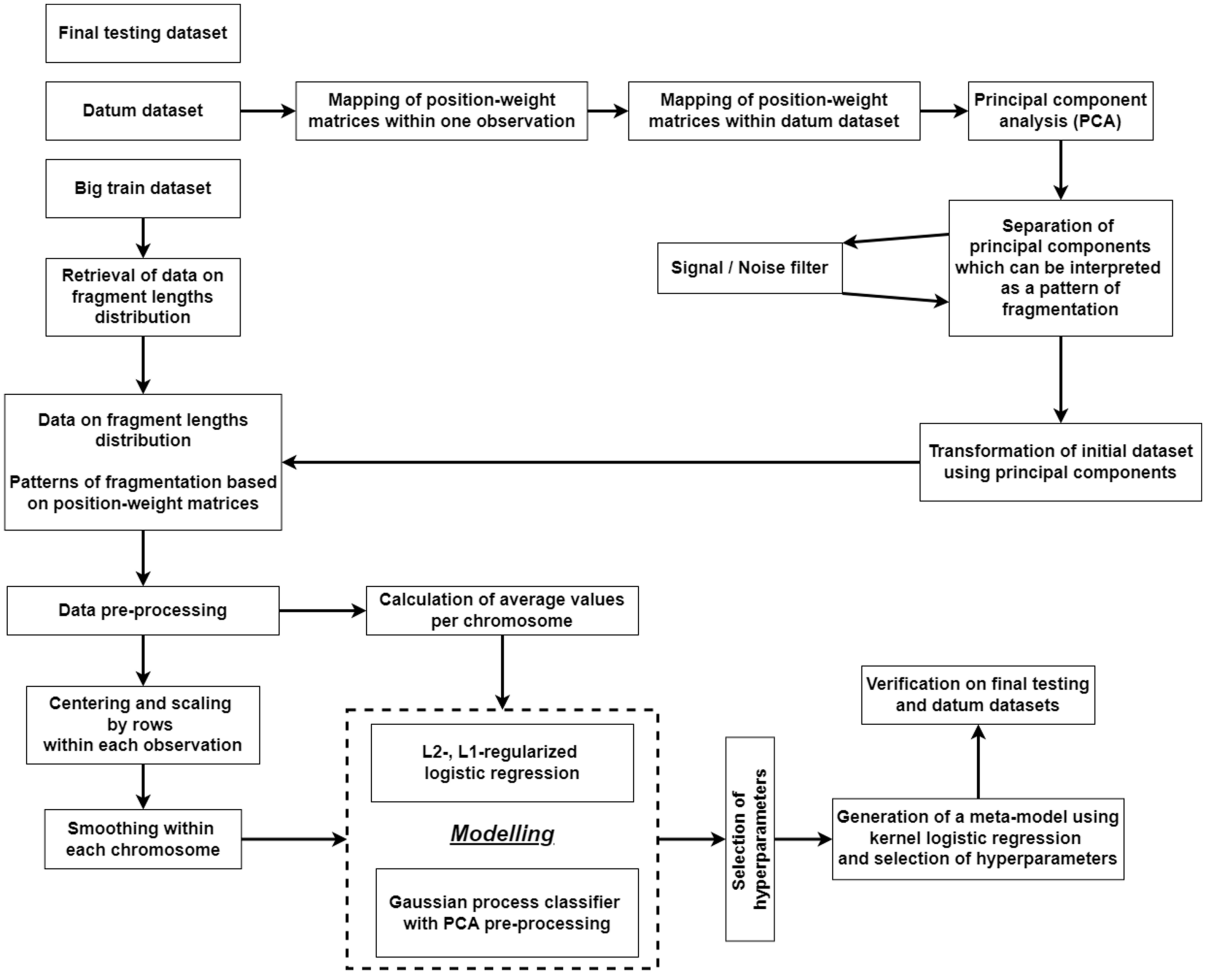
A flowchart of the algorithm for statistical processing of data on the cfDNA fragmentome.

The flowchart of the algorithm of statistical processing of converted NGS-data obtained after bioinformatics processing is presented in Figure 2.

Classification models of the first phase of machine learning were based either on logistic regression with L1- and L2-regularization or were probabilistic classification models based on Gaussian processes. The second-phase classification model was based on kernel logistic regression.

After calculating fragmentation patterns based on PWM values, an individual subject (or an observation) corresponds over 20,000 genomic intervals, each, in turn, is characterized by several variables. Thus, each observation is characterized by tens of thousands of variables, leading to the problem of high-dimensional data. Generation of a machine learning model with outstanding performance without applying methods for dimensionality reduction of the input data is difficult or even impossible. Therefore, before training the classification model, several methods like that were used. A different method of dimensionality reduction was used for each type of classifier.

Each logistic regression model captured only one characteristic of genomic intervals, such as average fragment length or fragmentation pattern. The values of a characteristic were averaged within each chromosome according to the formula (1):


(1) X¯i,chr=∑Jchrj=1xi,chrJchr,

where i – index of observation, chr – a chromosome name, Jchr – a number of genomic intervals within the chromosome chr , $\sum_{\mathrm{jchr}}^{j=1}x_{i,chr}$ – the sum of the values of the genome characteristic for all positions within the chromosome chr from initial to final Jchr .

After averaging, the number of input variables for the logistic regression model is reduced to 22 that corresponds to the number of human autosomes in the haploid set.

The result of logistic regression is the probability of the *i*-th subject belonging to the class of subjects with LC, which is calculated by the formula (2):


(2) $$p_{i}=\frac{1}{1+\exp\left(\beta_{0}+\beta^{T}X\right)}\text{,}$$

$$\beta_{0}+\beta^{T}X=\beta_{0}+\beta_{1}*{\bar{X}}_{i,\mathrm{metric},chr1}+\ldots +\beta_{22}*{\bar{X}}_{i,\mathrm{metric},chr22}$$

where $\beta_{0},\ldots ,\beta_{22}$ are coefficients, variables ${\bar{X}}_{i,\mathrm{metric},chr1},\ldots ,{\bar{X}}_{i,\mathrm{metric},chr22}$ are average values of a certain characteristic within each chromosome.

The calculation of coefficients $\beta_{0},\ldots ,\beta_{22}$ is performed by minimization of the loss function value $L\left(\beta_{0},\ldots ,\beta_{22},\lambda ,\alpha \right)$ , where λ and α – indications of hyperparameters.

Each Gaussian process classifier covers only one characteristic of genomic intervals. A flowchart of the procedure of dimensionality reduction of the input data for a Gaussian process classifier is shown in Supplementary Figure S3. As a result of this procedure, the dimension of the input data is reduced to several variables.

Gaussian process classifier allows one to calculate the probability of i -th subject belonging to the class of subjects with LC, indicated as $p_{i}$ , based on the training dataset and a set of predefined hyperparameters.

The formula (3) for calculation of $p_{i}$ is shown below:


(3) $$p_{i}=\Phi \left(\;\Psi_{\mathrm{training}},\psi_{i},\theta_{1},\theta_{2},\mathrm{stdz}\right)\text{,}$$

where i – index of observation from the test sample, $\Psi_{\mathrm{training}}$ – training dataset, $\psi_{i}$ – an observation from the test sample, $\theta_{1}$ , $\theta_{2}$ , stdz – indications of hyperparameters, Φ – a function for calculation of $p_{i}$ based on the argument values (29, 30).

The predictions of the first phase models were used as variables in the second phase of machine learning. The output of logistic regression and Gaussian process classifier is a probability. Next, this metric was converted into the logarithm of the odds ratio (OR) according to the formula (4):


(4) $$l_{\mathrm{odds}}=\log\left(\frac{p}{1-p}\right)\text{,}$$

The resulting value is not limited by any limits above or below, therefore, in order to avoid the appearance of infinite values, the log (OR) values were limited not to exceed │10│. The data for the second phase of machine learning model were summarized in a table with rows and columns corresponding to observations and predictions of the first phase models, respectively. This table was converted using PCA. The informative components obtained were used as input variables for the final classification model based on kernel logistic regression.

Kernel logistic regression allows one to calculate the probability of *i*-th subject belonging to the class of subjects with LC, indicated as $p_{i}$ , according to the formula (5):


(5) $$p_{i}=\Phi \left(\Psi_{\mathrm{training}},\psi_{i},\lambda ,\sigma^{2}\right)\text{,}$$

where i – index of observation from the test sample, $\Psi_{\mathrm{training}}$ – training dataset, $\psi_{i}$ – an observation from the test sample, λ and $\sigma^{2}$ – indications of hyperparameters, Φ – a function for calculation of $p_{i}$ based on the argument values.

The metric $p_{i}$ is a final result of the entire classification model. The physical interpretation of $p_{i}$ is that the closer the metric to 1 or to 0, the greater the confidence that a subject belongs to the class of subjects with LC or healthy subjects, respectively.

The training of all classification models of both the first and second phases was carried out solely on the training dataset with selection of hyperparameters by grid search and Monte Carlo cross-validation. The training dataset was randomly stratified into internal training and validation datasets with a ratio of 75 to 25%, respectively. The model was trained with a given set of hyperparameters on the internal training dataset, and its performance was tested on the validation dataset. Stratification took into account the status of a subject (a subject with LC or a healthy subject). For each type of classification model and, in turn, for each combination of hyperparameters, 100 random splits into internal training and validation datasets were carried out, followed by training and testing of the model. The area under the ROC curve (AUC) was used as a classification quality metric.

All statistical calculations were performed in the R programming language version 4.2.1, using RStudio environment v. 2022.02.3.492 for data analysis.

## 3 Results

148 healthy subjects and 138 subjects with confirmed LC were included in the study according to the criteria described in Materials and Methods section. After conducting NGS-analysis of cfDNA fragments isolated from the blood plasma, training, datum and testing datasets were generated for bioinformatics and statistical processing of NGS-data. Stratified selection made it possible to keep the distribution of subjects by age and gender in all three generated datasets the same as it was in the original dataset. Table 1 shows the number of subjects in the training, datum and testing dataset, as well as the distribution of subjects by age, gender and TNM classification.

**Table 1:** Characteristics of the training, datum and testing datasets.

Variable		Descriptive statistics of the datasets		
		Training dataset	Datum dataset	Testing dataset
Age (median, lower and upper quartiles)		63; (54.5 … 69)	62; (53.5 … 69)	63; (54.75 … 68.25)
The number of subjects in the age group	< 55 years	44 (25.1%)	14 (25.5%)	14 (25%)
	55–63 years	50 (28.6%)	15 (27.3%)	16 (28.6%)
	64–69 years	40 (22.9%)	13 (23.6%)	13 (23.2%)
	> 69 years	41 (23.4%)	13 (23.6%)	13 (23.2%)
The number of healthy subjects		89 (50.9%)	29 (52.7%)	30 (53.6%)
The number of subjects with lung cancer	Stage I	23 (13.1%)	7 (12.7%)	6 (10.7%)
	Stage II	29 (16.6%)	9 (16.4%)	10 (17.9%)
	Stage III	31 (17.7%)	10 (18.2%)	10 (17.9%)
	Stage IV	3 (1.7%)	–	–
The total number of subjects with lung cancer		86 (49.1%)	26 (47.3%)	26 (46.4%)
Gender	Male	90 (51.4%)	30 (54.5%)	28 (50%)
	Female	85 (48.6%)	25 (45.5%)	28 (50%)
Smoking status in the LC group	Smokers	36 (41.9%)	16 (61.5%)	15 (57.7%)
	Non-smokers	50 (58.1%)	10 (38.5%)	11 (42.3%)
Smoking index in the LC group (median, lower and upper quartiles)		0; (0 … 30)	25; (0 … 37.5)	25; (0 … 40)

All three datasets have been created from the original dataset by stratified random sampling method using age, gender and subject status as stratification variables. The smoking index is calculated as a product between the number of daily smoked cigarettes and the overall duration of smoking status in years divided by the a dimensional coefficient of 20.

Based on the datum dataset, which included 55 observations, an array with dimensions of 1,455,300 (26,460 × 55) rows and 40 columns was generated. This array was then transformed using PCA. Twenty-eight informative principal components were selected, representing more than 90% of total variance of the original dataset in multivariate space using a scree plot (Supplementary Figure S4). The remaining principal components were omitted due to their low-informative value. The selected principal components were further considered as fragmentation patterns reflecting the characteristics of terminal motifs of cfDNA fragments on a metagenomic scale. The fragmentation patterns calculation was carried out on all the genomic intervals regarding each subject of the training, datum and testing datasets. In other words, 40 variables corresponding to PWM elements were replaced by 28 fragmentation patterns at each genomic interval regardless of a subject.

To determine the status of a subject at the first phase of classification, 33 and 24 models were generated based on logistic regression with L1- and L2-regularization and Gaussian processes. Each first-phase model covered only one characteristic of genomic intervals. The logistic regression models used the distribution of cfDNA fragment lengths (mean, standard deviation, slope, and kurtosis), logarithms of the ratio of the abundance of short fragments to the abundance of long fragments, and fragmentation patterns as input data. Gaussian processes models used the abundance of cfDNA fragment lengths within genomic intervals, the distribution of cfDNA fragment lengths, and fragmentation patterns as input data (Table 2).

**Table 2:** Methods used for data pre-processing in this study.

Data pre-processing method		Calculation the mean value within each chromosome	Centering and scaling within one observation followed by smoothing and averaging of chromosomal rows
Abundance indicators of DNA fragments of genomic intervals	A number of short fragments	no	yes
	A number of long fragments	no	yes
	A total number of fragments	no	yes
	The derivative of the ratio “the number of short to the number of long fragments”	yes	yes
Statistical metrics	Average	yes	yes
	Standard deviation	yes	yes
	Skewness	yes	yes
	Kurtosis	yes	yes
	Standard error of the mean	no	yes
Fragmentation patterns based on information about the terminal motifs of DNA fragments	Principal components (from 1 to 28)	yes	yes
Machine learning method used		Logistic regression with L1- and L2-regularization	Probabilistic classification model based on Gaussian processes

One hundred AUC-values were obtained for each set of hyperparameter values when tested on validation datasets during the generation of an individual model. Descriptive statistics (median, lower and upper quartile) were calculated from this set of AUC-values. A set of hyperparameters values corresponding to the highest median AUC-value was recognized as the best set and was further used for generation of input data array for the second-phase model. The results of hyperparameters selection for the first-phase models are shown in Supplementary Tables S7, S8.

Some Gaussian process models using data on a particular fragmentation pattern as input data were excluded from consideration. It was impossible to generate a classification model that demonstrated any acceptable performance when tested on the validation dataset using such input data regardless of the hyperparameters values.

To generate the second-phase model, a training dataset consisting of 175 observations and 57 variables was used. Each variable corresponded to a result obtained by a particular first-phase classification model.

Prior to the generation of the second-phase model, exploratory data analysis (EDA) was carried out. The training dataset was transformed using the PCA. Two informative principal components (PC1 and PC2) were selected. They represented in total more than 40% of the variance of the original dataset in the space of variables of the training dataset. Statistically significant differences of values of principal components between healthy subjects and subjects with LC were found. These differences were the most noticeable in the case of PC1 (Kruskal-Wallis’s test *p*-value <10^−16^, size effect value η^2^ = 0.72). The remaining principal components were omitted due to their low-informative value.

**Figure 3:**
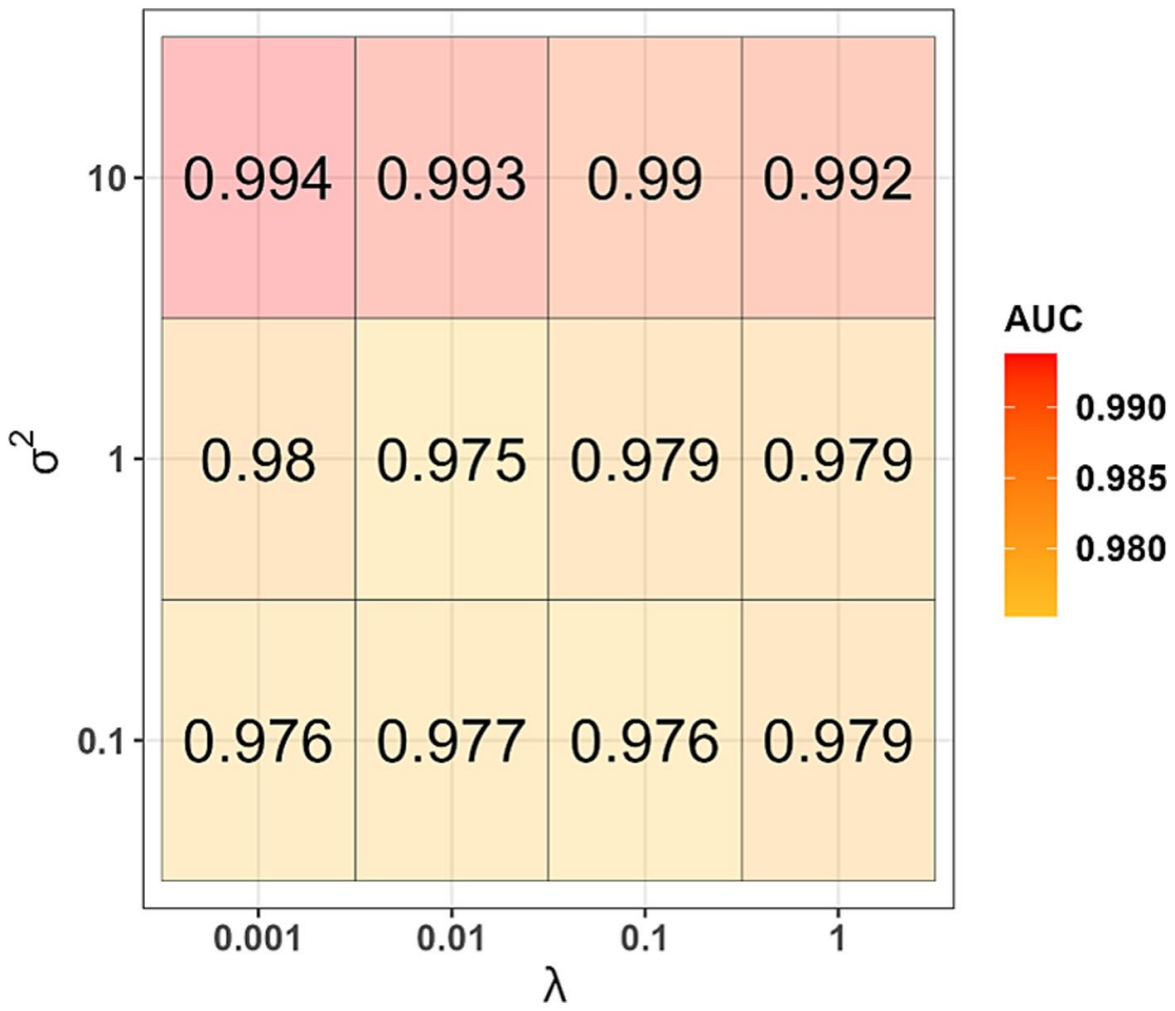
Results of selection of hyperparameters *λ* and σ2 (second phase of machine learning).

The informative principal components were used as independent variables to generate a kernel logistic regression model. During the model generation, for each set of values of the hyperparameters *λ* and σ^2^, 100 AUC-values were obtained when testing on validation datasets. The highest median AUC-value of 0.994 was obtained when using the hyperparameters values λ = 0.001 and σ^2^ = 10 (Figure 3). This set of hyperparameters values was found to be the best one. Using the entire training dataset as input data and the best values determined for the first- and second-phase models, we generated the final version of the classifier and evaluated its performance on the datum and testing datasets. AUC-values equal to 0.875 and 0.872 were obtained when tested on the datum and testing datasets, respectively.

The final classification model allows us to assess the probability of a subject with LC ( $p_{i}$ ), but does not unambiguously indicate whether the subject is healthy or with LC. Therefore, it was decided to determine the optimal threshold value for the $p_{i}$ -value.

**Figure 4:**
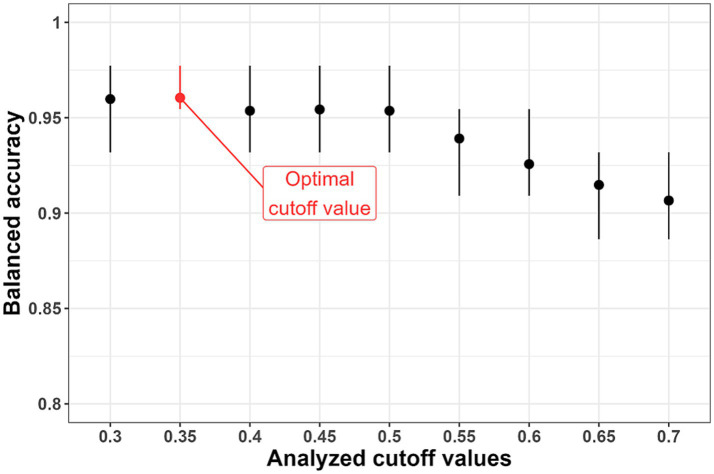
Results of selection of the threshold value ( $p_{i}$ -value). The optimal cutoff value is 0.35 due to the highest average values of balanced accuracy.

We conducted the following simulation experiment considering several options for the threshold value $p_{thr}$ for $p_{i}$ -value. For each option, 100 simulations, each consisting of several stages, were carried out. Briefly, the training dataset was divided into internal training and validation datasets. The internal training dataset was used to train the kernel logistic regression model with hyperparameters values *λ* = 0.001 and σ^2^ = 10, and the validation dataset was used to test the performance of this model. Subjects with $p_{i}$ ≥ $p_{thr}$ or $p_{i}$ < $p_{thr}$ were considered as subjects with LC or healthy subjects, respectively. Balanced accuracy was used as a classification quality metric. It was determined that the optimal $p_{thr}$ -value was equal to 0.35 according to the results of the simulation experiment (Figure 4).

**Figure 5:**
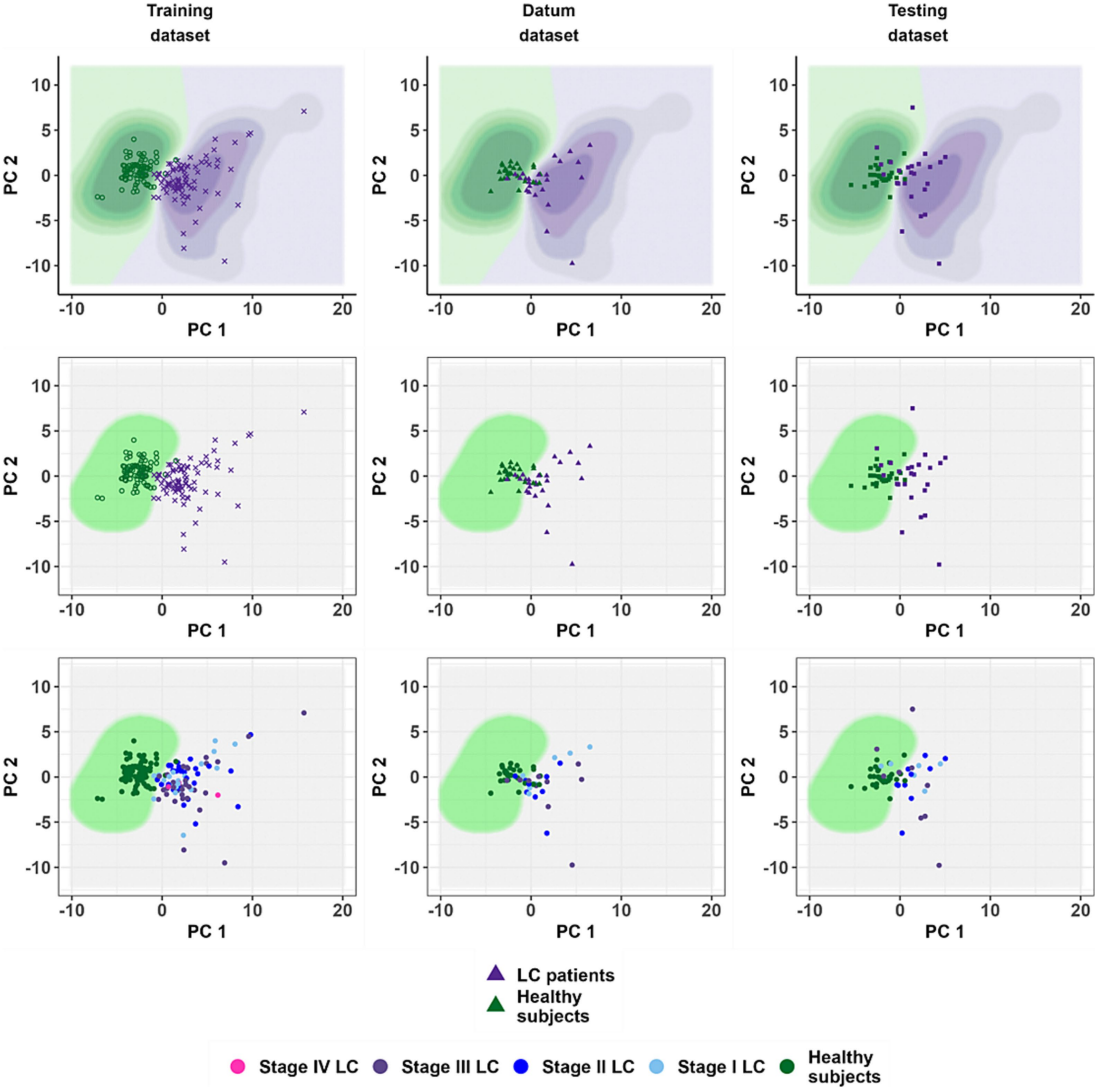
The location of the training dataset observations in the coordinates of the principal components and the result of testing the classification model on the datum and testing datasets. The coordinate axes forming the scatter diagram correspond to the principal components obtained during the calculation of the phase II model. The points correspond to patients. Panels from left to right: training, datum and testing dataset. The top row of panels shows the results obtained by the phase II model without using the cut-off value of 0.35. The middle row is a demonstration of the quality of the phase II model using a threshold value ( $p_{thr}$ ). The bottom row is a demonstration of the quality of performance of the phase II model in diagnosing lung cancer depending on the disease stages.

The sensitivity, specificity, and balanced accuracy of the final version of the classifier were evaluated when tested on the datum and testing datasets using this threshold value (Table 3; Figure 5). The sensitivity value when checking on the datum and testing datasets was 0.81, specificity − 0.79 and 0.90, respectively; balanced accuracy – 0.80 and 0.85, respectively.

**Table 3:** The classifier performance metrics calculated for training, datum and testing datasets.

Confusion matrices						
Model prediction	Real data					
	Training dataset		Datum dataset		Testing dataset	
	LC	H	LC	H	LC	H
Subjects with lung cancer (LC)	82	2	21	6	21	3
Healthy subjects (H)	4	87	5	23	5	27
Model’s performance metrics						
AUC	0.994		0.875		0.872	
Sensitivity	0.953		0.808		0.808	
Specificity	0.978		0.793		0.900	
Balanced accuracy	0.966		0.800		0.854	
Precision	0.976		0.778		0.875	
F1-measure	0.965		0.792		0.840	
Sensitivity for LC stage I	0.870		0.860		0.670	
Sensitivity for LC stage II	0.970		0.780		1.000	
Sensitivity for LC stage III	1.000		0.800		0.700	
Sensitivity for LC stage IV	1		–		–	

## 4 Discussion

The study demonstrated that NGS-analysis of cfDNA fragmentome isolated from blood plasma can be used to detect LC cases with high sensitivity and specificity. To our knowledge, it is the first cohort study with representative samples of healthy subjects and subjects with LC from Russian population. This can be considered as the first aspect of the novelty of the study. Another aspect of novelty is the unique combination of statistical and machine learning methods used to generate the binary classification model. Experimental part to obtain data on cfDNA fragmentome are consisted of highly reproducible methods (commercially available kits for cfDNA extraction, preparation of DNA-libraries and performing the Illumina NGS-sequencing).

Integration of data on the distribution of short and long cfDNA fragments (fragmentation pattern) and position-weight matrix data, reflecting end motifs of cfDNA fragments, resulted in high diagnostic performance of the classification model generated. This was achieved without additional data on mutational and methylome signatures of cfDNA, concentrations of proteomic biomarkers in blood plasma, clinical data of patients and radiological data. In fact, this points to the advantage of LC detection based solely on analysis of cfDNA fragments from blood plasma samples.

It should be noted about the limitations of the study. Thus, the cohort of subjects with confirmed LC was mixed by cancer stages. The cases with different stages of LC (I and II − early stages, III – advanced stage) were represented in approximately equal proportions in the testing and validation samples. In addition, several subjects with LC stage IV were represented in the training sample.

The generated method demonstrates the best sensitivity values when detecting LC stages II and III. Therefore, in this context, it is relevant to consider the applicability of the binary classification model for distinguishing healthy subjects and subjects with non-metastatic LC.

Another limitation is a lack of information about the performance of the binary classification model in relation to other cancer types. For example, using the DELFI machine learning method, which was originally adapted for the detection of LC, colorectal cancer (CRC) and hepatocellular carcinoma detection were demonstrated (25), as well as in the studies of monitoring patients with CRC (31) or liver cancer diagnostics (32). In this context, future studies with inclusion of subjects with different cancer types will reveal the specificity and areas of applicability of the approach for classification model design.

The results of the current study can be compared with other diagnostic methods based on NGS-analysis of cfDNA fragmentome. The classification model Lung-CLiP generated by Chabon and colleagues (33) uses input data on targeted sequencing of the same genomic intervals-of-interest of blood plasma cfDNA and leukocyte DNA. The Lung-CLiP covers the data on cfDNA fragments length distribution, single nucleotide variants and copy number variations. It has been shown that the Lung-CLiP yielded 16, 52 and 80% sensitivity in cases of 1 mL, 10 mL and > 10 mL sample volumes, respectively. The specificity values of the Lung-CLiP were 99 and 96% for the training and test cohorts, respectively (33).

The DELFI method presented by Cristiano and colleagues (21) is based on the analysis of low-coverage sequencing data of cfDNA fragmentome. The authors evaluated the length of each detected fragment and mapped it to the genomic origin. They divided the genome into non-overlapping intervals, each 5 million base pairs in length, and evaluated the distribution of fragment lengths for each interval. The method results in the DELFI metric that classifies a subject on to the healthy subject or subject with cancer. In total, 236 subjects with LC and 245 healthy subjects were included in the study (21). The DELFI method has been shown to have a sensitivity of 57 to 99% with a specificity of 98% depending on the cancer type. Mathios and colleagues (22) applied the DELFI method in a sample of 46 subjects with LC and 385 healthy subjects. It was shown that when choosing the cut-off value of the DELFI metric at 80% specificity, the values of diagnostic sensitivity are 57, 58 and 100% for LC stage I, II and III – IV, respectively. It has also been found that the DELFI metric can be used to analyze the survival rates of subjects with LC (22).

In the present work the performance of developed classification model was evaluated using datum and testing sets for each lung cancer stage category. The results of evaluation are presented in Table 3. Sensitivity values ranged from 66.7% to 85.7% for LC stage I, from 77.8 to 100% (stage II) and from 70 to 80% (stage III). Specificity values ranged from 79.3 to 90%. As can be seen, the performance metrics of classification solution developed in the present study are close or even identical to characteristics of diagnostic approaches applied by other research teams.

It should be noted that the cumulative findings on eight studies reviewed in the meta-analysis by He and colleagues (34) additionally support the validity of our classification model generated. They assessed the analytical accuracy of cfDNA-based biomarkers for the diagnosis of non-small cell LC. An average AUC value of 0.89 was found, which is very close to AUC values obtained in this work (0.87–0.875). It allows us to consider the classification model as corresponding to the analogs level and having a good diagnostic value.

The metrics such as sensitivity, specificity and AUC are crucial characteristics to prove the diagnostic performance of the classification model. In addition, the possibilities for clinical implementation of our classification model are indicated by the following advantages: (1) the performance of the classification model requires data exclusively from NGS-analysis of cfDNA fragmentome isolated from blood plasma and the accuracy of the classification model does not depend on the presence of other clinical data; (2) the classification model’s decision-making is interpretable and traceable; (3) modularity of the classification model.

First, the classification model does not take into consideration any variables that are derived from radiologic studies, biochemical and clinical analysis of blood samples and histology analysis of biopsy samples. One can note also an advantage of liquid biopsy approach: no traumatic and high-risk tissue biopsy procedure required.

Second, the classification model is consisted of several modules. Each module is relatively easily interpretable and may be represented as a logistic regression formula or as a sequence of matrix operations. Each module calculates the probability of LC presence using only a certain part of the indicators from the original set of 106,074 variables. Furthermore, the results given by each module can be are graphically represented as one- or two-dimensional scatter plot for the first and second phases of classification. As a matter of fact, this plot depicts one- or two-dimensional space where each of projected points correspond to subjects. The location of a point in one or another area of such a low-dimensional space corresponds to a greater or lesser probability of belonging to the group of subjects with LC.

The coordinate axes of the space are quantitative variables that, in turn, are calculated as a linear combinations of input data or through sigmoid transformation of such linear combinations. Therefore, there is the ability to trace the decisions made by all classification models of the 1st phase.

The resulted input data for the second phase classification model is two-dimensional matrix where rows are observations that correspond to subjects and columns are variables that correspond to probabilities of calculated by the models of the first phase. The observations are projecting to the low-dimensional space by the sequence of algebraic operations. The region of this space corresponds to the decision to classify the observation to the group of subjects with LC or to the group of healthy subjects. Our classification model presented as a set of formulae with supplementary one- and two-dimensional plots cannot be considered as a “black box” and is easier to interpret in comparison with trained neural networks and random forests (35).

If new characteristics of cfDNA fragments are added during modeling in cfDNA fragmentome studies, opportunities for creating of new modules will open up and, ultimately, it will contribute to improving the quality of the classification model. Moreover, the very architecture of model offers the ability to improve the classification performance by integrating the results of external diagnostical solutions. The computer vision system can analyze lung computed tomography (CT) scans and returns categorical or probabilistic value describing the status of the subject. For a cohort of participants, the resulted values can be incorporated in the architecture of classification model both at the first or at the second phases of machine learning as an input variable. These measures may yield better separation between analyzed classes but such modification requires new study cohort with genomic-wide characterization of cfDNA fragments and CT scan obtained for each study participant.

NGS-analysis of nucleic acids isolated from biospecimens produces a large amount of biological data (‘big data’), the processing and interpretation of which provide information to diagnose and clinical decision-making (36). The method for LC detection demonstrated in this work can become the basis for the development of a highly specific and minimally invasive test based on NGS-analysis of cfDNA for the early detection of malignant neoplasms. Thus, after clinical validation of the method, on the one hand, NGS-analysis of blood plasma cfDNA fragmentome may be an additional diagnostic option in the case of questionable and difficult interpretation of radiological data in order to minimize the need to perform a highly traumatic lung tissue biopsy or to verify the diagnosis in the case of biopsy failure of affected area. On the other hand, NGS-analysis of cfDNA fragmentome may in the future become a screening step for identifying suspected LC cases with further disease verification in accordance with the recommendations of national health authorities.

In conclusion, the described bioinformatics and statistical algorithm for NGS-data processing for minimally invasive diagnostics of LC stage I-III by blood plasma cfDNA allowed us to generate a binary classification model based on a unique combination of machine learning methods (L1–, L2 – regularized logistic regression, Gaussian process classifier, principal component method and kernel logistic regression). The classification model is characterized by AUC values from 0.872 to 0.875 (from 0.8 to 0.9 the quality is ‘excellent’) and makes it possible to distinguish healthy subjects and subjects with LC stage I-III by cfDNA fragmentation features. The classification model operates without using additional information about genomic, transcriptomic, proteomic and metabolomic biomarkers, clinical data of the subjects and the results of other instrumental techniques, including imaging techniques (radiography and computed tomography).

## Data Availability

The datasets presented in this article are not readily available because the dataset is a part of a proprietary algorithm and since it is not available due to legal restrictions. The computer code of this study is not publicly available since it is an implementation of a proprietary algorithm. An application for patenting an invention “Method for minimally invasive diagnosis of lung cancer using fragmented freely circulating DNA based on machine learning methods” to the Russian Federal Institute of Industrial Property, No. 2023120991, dated August 10, 2023 (the decision to grant a patent RU2023120991A dated May 02, 2024). Requests to access the datasets should be directed to IMeshkov@cspfmba.ru.

## References

[1] GLOBOCAN. Global Cancer Observatory. (2020). Available at: http://gco.iarc.fr/today/data/factsheets/cancers/15-Lung-fact-sheet.pdf (accessed on 22 March, 2024)

[2] Luo G Zhang Y Etxeberria J Arnold M Cai X Hao Y. Projections of lung cancer incidence by 2035 in 40 countries worldwide: population-based study. JMIR public Heal Surveill. (2023) 9:e43651. doi: 10.2196/43651, PMID: 36800235 PMC9984998

[3] Sharma R. Mapping of global, regional and national incidence, mortality and mortality-to-incidence ratio of lung cancer in 2020 and 2050. Int J Clin Oncol. (2022) 27:665–75. doi: 10.1007/s10147-021-02108-2, PMID: 35020103 PMC8753949

[4] Huang J Deng Y Tin MS Lok V Ngai CH Zhang L. Distribution, risk factors, and temporal trends for lung cancer incidence and mortality: a global analysis. Chest. (2022) 161:1101–11. doi: 10.1016/j.chest.2021.12.655, PMID: 35026300

[5] Sands J Tammemägi MC Couraud S Baldwin DR Borondy-Kitts A Yankelevitz D. Lung screening benefits and challenges: a review of the data and outline for implementation. J Thorac Oncol. (2021) 16:37–53. doi: 10.1016/j.jtho.2020.10.127, PMID: 33188913

[6] Marant-Micallef C Shield KD Vignat J Cléro E Kesminiene A Hill C. The risk of cancer attributable to diagnostic medical radiation: estimation for France in 2015. Int J Cancer. (2019) 144:2954–63. doi: 10.1002/ijc.32048, PMID: 30537057

[7] Dahal S Budoff MJ. Low-dose ionizing radiation and cancer risk: not so easy to tell. Quant Imaging Med Surg. (2019) 9:2023–6. doi: 10.21037/qims.2019.10.18, PMID: 31929978 PMC6942975

[8] Telekes A Horváth A. The role of cell-free DNA in cancer treatment decision making. Cancers (Basel). (2022) 14:611514. doi: 10.3390/cancers14246115, PMID: 36551600 PMC9776613

[9] Bao H Chen X Xiao Q Yang S Wu S Wang X. Associations of genome-wide cell-free DNA fragmentation profiles with blood biochemical and hematological parameters in healthy individuals. Genomics. (2022) 114:110504. doi: 10.1016/j.ygeno.2022.110504, PMID: 36257481

[10] Nikanjam M Kato S Kurzrock R. Liquid biopsy: current technology and clinical applications. J Hematol Oncol. (2022) 15:131. doi: 10.1186/s13045-022-01351-y, PMID: 36096847 PMC9465933

[11] Shields MD Chen K Dutcher G Patel I Pellini B. Making the rounds: exploring the role of circulating tumor DNA (ctDNA) in non-small cell lung cancer. Int J Mol Sci. (2022) 23:9006. doi: 10.3390/ijms23169006, PMID: 36012272 PMC9408840

[12] Liu Y. At the dawn: cell-free DNA fragmentomics and gene regulation. Br J Cancer. (2022) 126:379–90. doi: 10.1038/s41416-021-01635-z, PMID: 34815523 PMC8810841

[13] Thierry AR. Circulating DNA fragmentomics and cancer screening. Cell genomics. (2023) 3:100242. doi: 10.1016/j.xgen.2022.100242, PMID: 36777187 PMC9903826

[14] Joosse SA Pantel K. Circulating DNA and liquid biopsies in the management of patients with Cancer. Cancer Res. (2022) 82:2213–5. doi: 10.1158/0008-5472.CAN-22-1405, PMID: 35702889

[15] Kustanovich A Schwartz R Peretz T Grinshpun A. Life and death of circulating cell-free DNA. Cancer Biol Ther. (2019) 20:1057–67. doi: 10.1080/15384047.2019.1598759, PMID: 30990132 PMC6606043

[16] Avanzini S Kurtz DM Chabon JJ Moding EJ Hori SS Gambhir SS. A mathematical model of ctDNA shedding predicts tumor detection size. Sci Adv. (2020) 6:eabc4308. doi: 10.1126/sciadv.abc4308, PMID: 33310847 PMC7732186

[17] Pantel K Alix-Panabières C. Liquid biopsy and minimal residual disease - latest advances and implications for cure. Nat Rev Clin Oncol. (2019) 16:409–24. doi: 10.1038/s41571-019-0187-3, PMID: 30796368

[18] Mouliere F Robert B Arnau Peyrotte E Del Rio M Ychou M Molina F. High fragmentation characterizes tumour-derived circulating DNA. PLoS One. (2011) 6:e23418. doi: 10.1371/journal.pone.0023418, PMID: 21909401 PMC3167805

[19] Hallermayr A Wohlfrom T Steinke-Lange V Benet-Pagès A Scharf F Heitzer E. Somatic copy number alteration and fragmentation analysis in circulating tumor DNA for cancer screening and treatment monitoring in colorectal cancer patients. J Hematol Oncol. (2022) 15:125. doi: 10.1186/s13045-022-01342-z, PMID: 36056434 PMC9438339

[20] Pons-Belda OD Fernandez-Uriarte A Diamandis EP. Can circulating tumor dna support a successful screening test for early cancer detection? The grail paradigm. Diagnostics (Basel). (2021) 11:2171. doi: 10.3390/diagnostics11122171, PMID: 34943407 PMC8700281

[21] Cristiano S Leal A Phallen J Fiksel J Adleff V Bruhm DC. Genome-wide cell-free DNA fragmentation in patients with cancer. Nature. (2019) 570:385–9. doi: 10.1038/s41586-019-1272-6, PMID: 31142840 PMC6774252

[22] Mathios D Johansen JS Cristiano S Medina JE Phallen J Larsen KR. Detection and characterization of lung cancer using cell-free DNA fragmentomes. Nat Commun. (2021) 12:5060. doi: 10.1038/s41467-021-24994-w, PMID: 34417454 PMC8379179

[23] Guo W Chen X Liu R Liang N Ma Q Bao H. Sensitive detection of stage I lung adenocarcinoma using plasma cell-free DNA breakpoint motif profiling. EBioMedicine. (2022) 81:104131. doi: 10.1016/j.ebiom.2022.104131, PMID: 35780566 PMC9251329

[24] Wang S Meng F Li M Bao H Chen X Zhu M. Multidimensional cell-free DNA fragmentomic assay for detection of early-stage lung cancer. Am J Respir Crit Care Med. (2023) 207:1203–13. doi: 10.1164/rccm.202109-2019OC, PMID: 36346614 PMC10161762

[25] Bao H Wang Z Ma X Guo W Zhang X Tang W. Letter to the editor: an ultra-sensitive assay using cell-free DNA fragmentomics for multi-cancer early detection. Mol Cancer. (2022) 21:129. doi: 10.1186/s12943-022-01594-w, PMID: 35690859 PMC9188251

[26] Amemiya HM Kundaje A Boyle AP. The ENCODE blacklist: identification of problematic regions of the genome. Sci Rep. (2019) 9:9354. doi: 10.1038/s41598-019-45839-z, PMID: 31249361 PMC6597582

[27] Benjamini Y Speed TP. Summarizing and correcting the GC content bias in high-throughput sequencing. Nucleic Acids Res. (2012) 40:e72. doi: 10.1093/nar/gks001, PMID: 22323520 PMC3378858

[28] Claverie JM Audic S. The statistical significance of nucleotide position-weight matrix matches. Comput Appl Biosci. (1996) 12:431–9. doi: 10.1093/bioinformatics/12.5.431, PMID: 8996792

[29] Kuss M Rasmussen CE. Assessing approximate inference for binary Gaussian process classification. Mach Learn Res. (2005) 6:1679–704.

[30] Krasser M. (2020). Gaussian processes for classification. Available at: http://krasserm.github.io/2020/11/04/gaussian-processes-classification/ (Accessed November 18, 2023).

[31] Alipanahi B Lumbard KW Rinaldi L Carey J. Abstract 5714: cell-free DNA fragmentation profiling for monitoring therapeutic response in metastatic colorectal cancer. Cancer Res. (2023) 83:5714–4. doi: 10.1158/1538-7445.AM2023-5714

[32] Foda ZH Annapragada AV Boyapati K Bruhm DC Vulpescu NA Medina JE. Detecting liver cancer using cell-free DNA fragmentomes. Cancer Discov. (2023) 13:616–31. doi: 10.1158/2159-8290.CD-22-0659, PMID: 36399356 PMC9975663

[33] Chabon JJ Hamilton EG Kurtz DM Esfahani MS Moding EJ Stehr H. Integrating genomic features for non-invasive early lung cancer detection. Nature. (2020) 580:245–51. doi: 10.1038/s41586-020-2140-0, PMID: 32269342 PMC8230734

[34] He X Chi Y Peng J Hu W Ding C Li B. A systematic review and meta-analysis of circulating cell-free DNA as a diagnostic biomarker for non-small cell lung cancer. J Thorac Dis. (2022) 14:2103–11. doi: 10.21037/jtd-22-646, PMID: 35813759 PMC9264058

[35] Abdullah TAA Zahid MSM Ali W. A review of interpretable ML in healthcare: taxonomy, applications, challenges, and future directions. Symmetry (Basel). (2021) 13:2439. doi: 10.3390/sym13122439

[36] Jiang P Sinha S Aldape K Hannenhalli S Sahinalp C Ruppin E. Big data in basic and translational cancer research. Nat Rev Cancer. (2022) 22:625–39. doi: 10.1038/s41568-022-00502-0, PMID: 36064595 PMC9443637

