# Supplementary material for: Diagnostics of lung cancer by fragmentated blood circulating cell-free DNA based on machine learning methods
Source: Front Med (Lausanne). 2025 Jan 29;12:1435428. doi: 10.3389/fmed.2025.1435428 (PMC11813899; doi:10.3389/fmed.2025.1435428)
Supplement: Supplementary file 1 [file Data_Sheet_1.docx]

Diagnostics of lung cancer by fragmentated blood circulating cell-free DNA based on machine learning methods

Ivan O. Meshkov ^1^, Alexander P. Koturgin ^1^, Pavel V. Ershov ^1,^ *, Liubov A. Safonova ^1^, Julia A. Remizova ^1^, Valentina V. Maksyutina ^1^, Ekaterina D. Maralova ^1^, Vasilisa A. Astafieva ^1^, Alexey A. Ivashechkin ^1^, Boris D. Ignatiev ^1^, Antonida V. Makhotenko ^1^, Ekaterina A. Snigir ^1^, Valentin V. Makarov ^1^, Vladimir S. Yudin ^1^, Anton A. Keskinov ^1^, Sergey M. Yudin ^1^, Anna S. Makarova ^1^, Veronika I. Skvortsova ^2^

^1^ Federal State Budgetary Institution “Centre for Strategic Planning and Management of Biomedical Health Risks” of the Federal medical and biological agency (Centre for Strategic Planning, of the Federal medical and biological agency), 10 bld. 1 Pogodinskaya Str., Moscow, 119121, Russia

2 The Federal medical and biological agency (FMBA of Russia), Volokolamskoye shosse, 30, 123182, Moscow, Russia

***** Correspondence: IMeshkov@cspfmba.ru

***Supplementary materials***

**Supplementary material to the subsection 2.9.2 of “Materials and Methods” section**

**Gaussian process classification model generation**

Each Gaussian process classifier covered single characteristic of genomic intervals. In turn, the characteristic value was determined for each genomic interval. Thus, several thousand variables corresponded to one characteristic, for example, the number of short fragments or the fragmentation pattern.

This data array can be represented according to S1:

$X=\left\{ x_{i, chr1,1},x_{i, chr1,2},\ldots,x_{i,chrr,k}\ldots,x_{i,chr22,j} \right\}$ (S1),

where *i* – the index of a subject, that is an observation; *j* – the number of genomic intervals; *chr1*, *chr2*, … *chr22* – the chromosome indications to which a particular genomic interval belongs; $x_{i,chrr,k}$ – the characteristic value on the genomic interval *k* located on chromosome *r*.

Within each observation, the median value and interquartile range of the characteristic across the entire genome were calculated. Then the values contained in the data array were converted according to the formula S2:

$u_{ij}= \frac{x_{ij}-{Med}_{i}}{{IQR}_{i}}$ (S2),

where *i* – the index of a subject; ${Med}_{i}$ – the median characteristic value calculated across the entire genome of the subject *i*; ${IQR}_{i}$ – the interquartile range of the characteristic calculated across the entire genome of the subject *i*.

Then, whole data array $U=\left\{ u_{i, 1},\ldots,u_{i,j} \right\}$ was divided into 22 separate tables, according to the number of chromosomes. For each subject, next two operations were carried out separately and within each table:

1) For a number of values $U=\{u_{i, 1},\ldots,u_{i,j}\}$, a sliding average with a window width of two genomic intervals was passed several times. Consistently, sliding smoothing was carried out several times. Each time, after passing the sliding average, the length of sets became shorter by one value. When the length of a set became a factor of ten, the sliding average was stopped.

2) The smoothed set was divided into non-overlapping intervals of ten values wide. Since the length of the smoothed set is a factor of ten, it is completely covered by these non-overlapping intervals. Within each interval, the average was calculated. The resulting averages represented a new set of values, which looked like “convolution” of its predecessor. The transformed series were combined into a new set of variables $V$ according to S3:

$V=\{v_{i, chr1,1},v_{i, chr1,2},\ldots,v_{i,chr22,w}\}$ (S3),

where *i* – the index of a subject; *w* – the final length of a set of variables that was obtained during the transformation.

3) A set of variables *V* was transformed using the principal component analysis with subsequent selection of informative principal components (PC).

4) Informative PCs obtained were used as input variables for the probabilistic Gaussian process classifier.

The probability of the *i-th* subject belonging to the class of subjects with lung cancer (LC) was calculated using this classifier according to the formula S4:

$p_{i}= \Phi({\Psi_{training}, \psi_{i},\theta}_{1},\theta_{2},stdz)$ (S4),

where $p_{i}$ – the probability of the *i-th* subject belonging to the class of subjects with LC; $\Psi_{training}$ – the training dataset; $\psi_{i}$ – the observation of test dataset; $\theta_{1}$, $\theta_{2}$, $stdz$ – indications of hyperparameters; $\Phi$ – the function for calculation of $p_{i}$ on the basis of arguments values.

The optimal values of the hyperparameters $\theta_{1}$, $\theta_{2}$ and $sdtz$ were selected during cross-validation. Hyperparameters $\theta_{1}$, $\theta_{2}$ are included in calculation of the values of the radial basis function and play a key role in the probabilistic classification algorithm. The hyperparameter $sdtz$ denotes centering and scaling of the input data before generation of a probabilistic classifier: “0” or “1” means that centering and scaling were not carried out or carried out, respectively. Centering and scaling were carried out according to the formula S5:

$\psi_{ij}'= \frac{\psi_{ij}-Med(\Psi_{training,j})}{IQR(\Psi_{training,j})}$ (S5),

where $i$ – the index of input observation; $j$ – the index of the variable describing the input observation (the index of informative PC); $Med\left( \Psi_{training,j} \right)$ – the median value of the variable $j$ calculated using the training dataset; $IQR(\Psi_{training,j})$ – the interquartile range of variable $j$ calculated using the training dataset; $\psi_{ij}$ – the observation of test dataset; $\psi_{ij}'$ – the observation of test dataset transformed by centering and scaling.

**Table S1.** Clinical characteristics of the healthy subjects in the training dataset

| **ID** | **Age** | **Gender** | **Race** |
| --- | --- | --- | --- |
| 78003091 | 59 | Female | Caucasian |
| 78003018 | 55 | Male | Caucasian |
| 78003026 | 60 | Male | Caucasian |
| 78003033 | 64 | Male | Caucasian |
| 78003030 | 64 | Male | Caucasian |
| 78003035 | 65 | Male | Caucasian |
| 78003013 | 71 | Male | Caucasian |
| 78003010 | 69 | Male | Caucasian |
| 78003009 | 52 | Male | Caucasian |
| 78003023 | 75 | Male | Caucasian |
| 78003024 | 71 | Male | Caucasian |
| 78003025 | 73 | Male | Caucasian |
| 78003048 | 71 | Female | Caucasian |
| 78003050 | 74 | Female | Caucasian |
| 78003051 | 71 | Female | Caucasian |
| 78003052 | 57 | Female | Caucasian |
| 78003053 | 65 | Female | Caucasian |
| 78003055 | 66 | Female | Caucasian |
| 78003057 | 71 | Female | Caucasian |
| 78003058 | 71 | Female | Caucasian |
| 78003059 | 65 | Female | Caucasian |
| 78003060 | 50 | Female | Caucasian |
| 78003061 | 59 | Female | Caucasian |
| 78003062 | 77 | Female | Caucasian |
| 78003066 | 67 | Female | Caucasian |
| 78003067 | 70 | Female | Caucasian |
| 78003073 | 55 | Female | Caucasian |
| 78003074 | 62 | Female | Caucasian |
| 78003076 | 66 | Female | Caucasian |
| 78003081 | 62 | Female | Caucasian |
| 78003084 | 63 | Female | Caucasian |
| 78003085 | 58 | Female | Caucasian |
| 78003088 | 58 | Female | Caucasian |
| 78003092 | 65 | Female | Caucasian |
| 78003093 | 69 | Female | Caucasian |
| 78003094 | 53 | Female | Caucasian |
| 78003095 | 72 | Female | Caucasian |
| 78003001 | 63 | Male | Caucasian |
| 78003019 | 70 | Male | Caucasian |
| 78003020 | 66 | Male | Caucasian |
| 78003008 | 59 | Male | Caucasian |
| 78003007 | 59 | Male | Caucasian |
| 78003000 | 71 | Male | Caucasian |
| 78003004 | 56 | Male | Caucasian |
| 78003012 | 61 | Male | Caucasian |
| 78003014 | 60 | Male | Caucasian |
| 78003027 | 73 | Male | Caucasian |
| 78003896 | 43 | Male | Caucasian |
| 78003900 | 41 | Female | Caucasian |
| 78003903 | 45 | Male | Caucasian |
| 78003904 | 59 | Female | Caucasian |
| 78003905 | 54 | Male | Caucasian |
| 78003908 | 63 | Male | Caucasian |
| 78003920 | 68 | Female | Caucasian |
| 78003922 | 41 | Male | Caucasian |
| 78003923 | 68 | Male | Caucasian |
| 78003925 | 69 | Male | Caucasian |
| 78003926 | 55 | Male | Caucasian |
| 78003927 | 66 | Male | Caucasian |
| 78003930 | 47 | Female | Caucasian |
| 78003932 | 42 | Female | Caucasian |
| 78003933 | 58 | Female | Caucasian |
| 78003934 | 68 | Female | Caucasian |
| 78003935 | 41 | Male | Caucasian |
| 78003936 | 60 | Female | Caucasian |
| 78003941 | 69 | Female | Caucasian |
| 78003943 | 47 | Male | Caucasian |
| 78003944 | 46 | Male | Caucasian |
| 78003945 | 44 | Male | Caucasian |
| 78003949 | 43 | Female | Caucasian |
| 78003950 | 50 | Male | Caucasian |
| 78003952 | 52 | Male | Caucasian |
| 78003955 | 76 | Male | Caucasian |
| 78003957 | 75 | Male | Caucasian |
| 78003958 | 59 | Female | Caucasian |
| 78003959 | 40 | Male | Caucasian |
| 78003972 | 59 | Female | Caucasian |
| 78003975 | 62 | Female | Caucasian |
| 78003976 | 46 | Female | Caucasian |
| 78003979 | 51 | Female | Caucasian |
| 78003982 | 62 | Female | Caucasian |
| 78003984 | 53 | Female | Caucasian |
| 78003985 | 45 | Male | Caucasian |
| 78003986 | 70 | Female | Caucasian |
| 78003987 | 43 | Male | Caucasian |
| 78003988 | 59 | Female | Caucasian |
| 78003990 | 55 | Female | Caucasian |
| 78003993 | 42 | Female | Caucasian |
| 78004002 | 57 | Male | Caucasian |

**Table S2.** Clinical characteristics of the healthy subjects in the datum dataset.

| **ID** | **Age** | **Gender** | **Race** |
| --- | --- | --- | --- |
| 78003029 | 71 | Male | Caucasian |
| 78003031 | 60 | Male | Caucasian |
| 78003038 | 58 | Male | Caucasian |
| 78003028 | 62 | Male | Caucasian |
| 78003043 | 62 | Female | Caucasian |
| 78003054 | 55 | Female | Caucasian |
| 78003063 | 60 | Female | Caucasian |
| 78003065 | 68 | Female | Caucasian |
| 78003068 | 67 | Female | Caucasian |
| 78003069 | 71 | Female | Caucasian |
| 78003072 | 76 | Female | Caucasian |
| 78003078 | 71 | Female | Caucasian |
| 78003079 | 56 | Female | Caucasian |
| 78003083 | 58 | Female | Caucasian |
| 78003090 | 52 | Female | Caucasian |
| 78003096 | 67 | Female | Caucasian |
| 78003097 | 51 | Female | Caucasian |
| 78003928 | 64 | Male | Caucasian |
| 78003946 | 43 | Male | Caucasian |
| 78003947 | 43 | Male | Caucasian |
| 78003956 | 52 | Male | Caucasian |
| 78003960 | 59 | Male | Caucasian |
| 78003962 | 70 | Male | Caucasian |
| 78003965 | 61 | Female | Caucasian |
| 78003989 | 42 | Female | Caucasian |
| 78003992 | 51 | Male | Caucasian |
| 78003002 | 65 | Male | Caucasian |
| 78003006 | 75 | Male | Caucasian |
| 78003005 | 69 | Male | Caucasian |

**Table S3.** Clinical characteristics of the healthy subjects in the testing dataset.

| **ID** | **Age** | **Gender** | **Race** |
| --- | --- | --- | --- |
| 78003021 | 58 | Male | Caucasian |
| 78003015 | 71 | Male | Caucasian |
| 78003034 | 56 | Male | Caucasian |
| 78003032 | 67 | Male | Caucasian |
| 78003011 | 51 | Male | Caucasian |
| 78003064 | 68 | Female | Caucasian |
| 78003071 | 73 | Female | Caucasian |
| 78003075 | 66 | Female | Caucasian |
| 78003077 | 60 | Female | Caucasian |
| 78003080 | 54 | Female | Caucasian |
| 78003082 | 60 | Female | Caucasian |
| 78003086 | 54 | Female | Caucasian |
| 78003087 | 67 | Female | Caucasian |
| 78003089 | 58 | Female | Caucasian |
| 78003091 | 59 | Female | Caucasian |
| 78003098 | 63 | Female | Caucasian |
| 78003099 | 74 | Female | Caucasian |
| 78003895 | 43 | Male | Caucasian |
| 78003899 | 64 | Male | Caucasian |
| 78003924 | 62 | Male | Caucasian |
| 78003942 | 66 | Female | Caucasian |
| 78003948 | 43 | Male | Caucasian |
| 78003951 | 71 | Female | Caucasian |
| 78003980 | 62 | Female | Caucasian |
| 78003983 | 41 | Male | Caucasian |
| 78003995 | 52 | Female | Caucasian |
| 78003996 | 48 | Female | Caucasian |
| 78003016 | 55 | Male | Caucasian |
| 78003003 | 71 | Male | Caucasian |
| 78003022 | 72 | Male | Caucasian |

**Table S4.** Clinical characteristics of the subjects with LC in the training dataset

| **ID** | **Age** | **Gender** | **Race** | **ICD-10 code** | **Stage** |
| --- | --- | --- | --- | --- | --- |
| 78003202 | 73 | Female | Caucasian | C34.15 | IA |
| 78003204 | 53 | Female | Caucasian | C34.3 | IIA |
| 78003205 | 64 | Female | Caucasian | С34.2 | IА3 |
| 78003208 | 55 | Male | Caucasian | C34.8 | IIIB |
| 78003209 | 73 | Female | Caucasian | С34.3 | IB |
| 78003210 | 70 | Female | Caucasian | С34.3 | IB |
| 78003214 | 77 | Female | Caucasian | С34.3 | IB |
| 78003216 | 74 | Male | Caucasian | С34.9 | IIIA |
| 78003273 | 36 | Female | Caucasian | С34.8 | IIB |
| 78003289 | 72 | Male | Caucasian | С34.8 | III |
| 78003295 | 54 | Male | Caucasian | C34.8 | IIB |
| 78003677 | 55 | Male | Caucasian | C34.1 | IB |
| 78003683 | 67 | Male | Caucasian | C34.1 | IIB |
| 78003685 | 48 | Male | Caucasian | C34.1 | IB |
| 78003690 | 65 | Female | Caucasian | C34.1 | IA2 |
| 78003693 | 66 | Female | Caucasian | С34.1 | IIIA |
| 78003696 | 76 | Female | Caucasian | C34.3 | IIA |
| 78003698 | 65 | Male | Caucasian | C34.3 | IB |
| 78003700 | 50 | Male | Caucasian | C34.3 | IIA3 |
| 78003705 | 70 | Male | Caucasian | C34.1 | IIA2 |
| 78003707 | 65 | Male | Caucasian | C34.1 | IIB |
| 78003710 | 48 | Female | Caucasian | C34.1 | IIIA |
| 78003711 | 68 | Female | Caucasian | C34.3 | IIIA |
| 78003712 | 74 | Male | Caucasian | C34.3 | IIIA |
| 78003713 | 60 | Male | Caucasian | C34.2 | IIA |
| 78003714 | 62 | Male | Caucasian | C34.1 | IIIA |
| 78003715 | 43 | Female | Caucasian | C34.1 | IIA |
| 78003716 | 62 | Male | Caucasian | C34 | IIB |
| 78003719 | 64 | Male | Caucasian | C34.1 | IIIA |
| 78003721 | 67 | Female | Caucasian | C34.3 | IB |
| 78003724 | 71 | Male | Caucasian | C34.1 | IIIB |
| 78003726 | 60 | Male | Caucasian | C34.1 | IIB |
| 78003728 | 50 | Male | Caucasian | C34.1 | IIIA |
| 78003729 | 84 | Male | Caucasian | C34.1 | IIB |
| 78003730 | 41 | Male | Caucasian | C34.1 | IIIB |
| 78003731 | 75 | Female | Caucasian | C34.3 | IIIA |
| 78003733 | 64 | Female | Caucasian | C34.3 | IA1 |
| 78003735 | 76 | Male | Caucasian | C34.3 | IIIC |
| 78003736 | 66 | Male | Caucasian | C34.3 | IIB |
| 78003742 | 70 | Female | Caucasian | C34.1 | IA2 |
| 78003745 | 67 | Female | Caucasian | C34.1 | IA |
| 78003746 | 63 | Female | Caucasian | C34.3 | IIIA |
| 78003747 | 52 | Female | Caucasian | C34.1 | IIB |
| 78003748 | 65 | Male | Caucasian | C34.1 | IIB |
| 78003749 | 57 | Female | Caucasian | C34.1 | IA2 |
| 78003752 | 58 | Male | Caucasian | C34.3 | IIIA |
| 78003753 | 62 | Male | Caucasian | C34.1 | IIB |
| 78003756 | 77 | Male | Caucasian | C34.1 | IIIA |
| 78003757 | 53 | Male | Caucasian | C34.1 | IA2 |
| 78003759 | 74 | Male | Caucasian | C34.3 | IA |
| 78003760 | 59 | Male | Caucasian | C34.3 | IIB |
| 78003762 | 54 | Male | Caucasian | C34.1 | IIB |
| 78003763 | 44 | Male | Caucasian | C34.1 | IIB |
| 78003767 | 58 | Female | Caucasian | C34.1 | IIIA |
| 78003771 | 56 | Male | Caucasian | C34.1 | IIB |
| 78003772 | 65 | Female | Caucasian | C34.1 | IB |
| 78003773 | 35 | Female | Caucasian | C34.1 | IB |
| 78003777 | 69 | Female | Caucasian | C34.1 | IIIA |
| 78003779 | 67 | Male | Caucasian | C34.3 | IIIA |
| 78003780 | 71 | Male | Caucasian | C34.1 | IIIA |
| 78003782 | 64 | Male | Caucasian | C34.2 | IB |
| 78003222 | 70 | Male | Caucasian | С34.8 | IIIА |
| 78003224 | 59 | Male | Caucasian | С34.8 | IIА |
| 78003264 | 67 | Male | Caucasian | С34.8 | IIIА |
| 78003258 | 63 | Male | Caucasian | C34.8 | IIA |
| 78003251 | 54 | Female | Caucasian | C34.8 | IA2 |
| 78003266 | 66 | Female | Caucasian | С34.8 | IIА |
| 78003253 | 51 | Male | Caucasian | С34.8 | IIIА |
| 78003254 | 53 | Male | Caucasian | C34.8 | IIIA |
| 78003255 | 53 | Female | Caucasian | C34.8 | IIIA |
| 78003265 | 54 | Male | Caucasian | С34.8 | IIIА |
| 78003268 | 67 | Female | Caucasian | С34.8 | IIIА |
| 78003221 | 52 | Female | Caucasian | C34.3 | IB |
| 78003220 | 67 | Female | Caucasian | C34.3 | IIB |
| 78003245 | 61 | Male | Caucasian | C34.1 | IIIB |
| 78003244 | 63 | Male | Caucasian | C34.8 | IVА |
| 78003235 | 69 | Male | Caucasian | C34.8 | IIIB |
| 78003234 | 61 | Female | Caucasian | C34.8 | I |
| 78003228 | 71 | Female | Caucasian | C34.8 | IIIA |
| 78003246 | 57 | Female | Caucasian | C34.8 | IIIА |
| 78003287 | 50 | Male | Caucasian | C34.8 | IIB |
| 78003263 | 63 | Male | Caucasian | С34.1 | IA2 |
| 78003240 | 58 | Female | Caucasian | С34.3 | IVB |
| 78003237 | 75 | Female | Caucasian | С34.1 | IVA |
| 78003271 | 71 | Female | Caucasian | С34.3 | IA |
| 78003262 | 76 | Female | Caucasian | С34.3 | I |

**Table S5.** Clinical characteristics of the subjects with LC in the datum dataset.

| **ID** | **Age** | **Gender** | **Race** | **ICD-10 code** | **Stage** |
| --- | --- | --- | --- | --- | --- |
| 78003207 | 70 | Male | Caucasian | C34.8 | IIB |
| 78003212 | 56 | Female | Caucasian | С34.8 | IA3 |
| 78003261 | 72 | Male | Caucasian | С34.3 | IIIB |
| 78003278 | 67 | Female | Caucasian | С34.1 | IB |
| 78003676 | 87 | Male | Caucasian | C34.3 | IIIA |
| 78003680 | 48 | Male | Caucasian | C34.3 | IIIA |
| 78003706 | 66 | Female | Caucasian | C34.0 | IIIB |
| 78003717 | 61 | Male | Caucasian | C34.3 | IIB |
| 78003725 | 74 | Female | Caucasian | C34.2 | IB |
| 78003737 | 50 | Male | Caucasian | C34.1 | IIIA |
| 78003744 | 51 | Female | Caucasian | C34.3 | IA3 |
| 78003751 | 62 | Male | Caucasian | C34.3 | IIB |
| 78003754 | 48 | Male | Caucasian | C34.3 | IA2 |
| 78003758 | 74 | Male | Caucasian | C34.1 | IIIA |
| 78003765 | 66 | Female | Caucasian | C34.3 | IB |
| 78003768 | 44 | Male | Caucasian | C34.3 | IIB |
| 78003769 | 32 | Male | Caucasian | C34.1 | IIB |
| 78003778 | 69 | Male | Caucasian | C34.1 | IIB |
| 78003781 | 68 | Male | Caucasian | C34.1 | IIIA |
| 78003233 | 47 | Female | Caucasian | C34.8 | IIB |
| 78003223 | 65 | Male | Caucasian | С34.8 | IIB |
| 78003226 | 63 | Male | Caucasian | С34.8 | IIIА |
| 78003249 | 71 | Female | Caucasian | C34.8 | IIB |
| 78003250 | 69 | Male | Caucasian | C34.8 | IIIA |
| 78003272 | 57 | Female | Caucasian | C34.3 | IIIC |
| 78003259 | 75 | Female | Caucasian | C34.1 | IB |

**Table S6.** Clinical characteristics of the subjects with LC in the testing dataset.

| **ID** | **Age** | **Gender** | **Race** | **ICD-10 code** | **Stage** |
| --- | --- | --- | --- | --- | --- |
| 78003276 | 67 | Female | Caucasian | С34.8 | IIIА |
| 78003671 | 83 | Female | Caucasian | C34.3 | IA |
| 78003672 | 63 | Female | Caucasian | C34.3 | IA2 |
| 78003673 | 67 | Female | Caucasian | C34.1 | IB |
| 78003674 | 58 | Male | Caucasian | C34.2 | IIB |
| 78003682 | 78 | Female | Caucasian | C34.1 | IIA3 |
| 78003687 | 50 | Male | Caucasian | C34.1 | IIIA |
| 78003701 | 74 | Female | Caucasian | C34.1 | IIA |
| 78003708 | 71 | Male | Caucasian | C34.3 | IIB |
| 78003722 | 68 | Male | Caucasian | C34.3 | IIA |
| 78003723 | 58 | Female | Caucasian | C34.0 | IIIA |
| 78003732 | 54 | Male | Caucasian | C34.3 | IIB |
| 78003750 | 44 | Female | Caucasian | C34.3 | IIB |
| 78003755 | 73 | Female | Caucasian | C34.3 | IB |
| 78003761 | 43 | Male | Caucasian | C34.3 | IIB |
| 78003774 | 53 | Male | Caucasian | C34.1 | IIIA |
| 78003776 | 66 | Male | Caucasian | C34.1 | IIIA |
| 78003213 | 69 | Female | Caucasian | С34.8 | IA3 |
| 78003227 | 61 | Male | Caucasian | С34.8 | IIIB |
| 78003256 | 65 | Male | Caucasian | C34.8 | IIB |
| 78003275 | 63 | Male | Caucasian | С34.8 | IIB |
| 78003248 | 76 | Male | Caucasian | C34.8 | IIIB |
| 78003229 | 66 | Male | Caucasian | С34.3 | III |
| 78003239 | 59 | Male | Caucasian | С34.0 | III |
| 78003260 | 71 | Male | Caucasian | С34.3 | IIIA |
| 78003270 | 53 | Female | Caucasian | С34.3 | IB |

**Table S7**. Selection of hyperparameters for first-stage models based on logistic regression with L1- and L2-regularization.

The hyperparameter *α* controls the balance between L1- and L2-regularization. The value *α* =1 or *α*=0 means that only L1- or L2-regularization is used, respectively. The value *λ*=0 means that logistic regression model is estimated without any regularization so *α*-value is not taken into consideration.

| Variables | Hyperparameter | | AUC value (median; [lower … upper quartile]) |
| --- | --- | --- | --- |
|  | *α* | *λ* |  |
| The average fragment length | 0.75 | 0.005 | 0.628; [0.558 ... 0.669] |
| Standard deviation of the fragment length distribution | - | 0 | 0.607; [0.545 ... 0.66] |
| Skew of the fragment length distribution | 1 | 0.005 | 0.618; [0.552 ... 0.655] |
| Kurtosis of the fragment length distribution | - | 0 | 0.604; [0.56 ... 0.639] |
| The derivative of the ratio “the number of short to the number of long fragments” | 0 | 0.005 | 0.631; [0.595 ... 0.675] |
| Fragmentation pattern 1 | 0 | 0.05 | 0.65; [0.592 ... 0.694] |
| Fragmentation pattern 2 | 0.1 | 0.0005 | 0.664; [0.617 ... 0.713] |
| Fragmentation pattern 3 | 0.25 | 0.005 | 0.68; [0.624 ... 0.719] |
| Fragmentation pattern 4 | 0.5 | 0.01 | 0.6; [0.551 ... 0.645] |
| Fragmentation pattern 5 | 0.75 | 0.005 | 0.813; [0.773 ... 0.846] |
| Fragmentation pattern 6 | 0.9 | 0.01 | 0.789; [0.749 ... 0.831] |
| Fragmentation pattern 7 | 0.1 | 0.5 | 0.598; [0.543 ... 0.635] |
| Fragmentation pattern 8 | 0.25 | 0.1 | 0.652; [0.595 ... 0.695] |
| Fragmentation pattern 9 | 0.25 | 0.005 | 0.777; [0.725 ... 0.818] |
| Fragmentation pattern 10 | - | 0 | 0.565; [0.529 ... 0.601] |
| Fragmentation pattern 11 | - | 0 | 0.56; [0.52 ... 0.591] |
| Fragmentation pattern 12 | 0.1 | 0.5 | 0.615; [0.562 ... 0.662] |
| Fragmentation pattern 13 | 0 | 0.005 | 0.562; [0.516 ... 0.595] |
| Fragmentation pattern 14 | 0.9 | 0.01 | 0.595; [0.541 ... 0.624] |
| Fragmentation pattern 15 | 1 | 0.005 | 0.748; [0.705 ... 0.798] |
| Fragmentation pattern 16 | 0.75 | 0.05 | 0.788; [0.747 ... 0.816] |
| Fragmentation pattern 17 | 0 | 0.5 | 0.594; [0.526 ... 0.636] |
| Fragmentation pattern 18 | - | 0 | 0.561; [0.52 ... 0.593] |
| Fragmentation pattern 19 | 0.5 | 0.01 | 0.585; [0.54 ... 0.625] |
| Fragmentation pattern 20 | 0.1 | 0.1 | 0.705; [0.648 ... 0.744] |
| Fragmentation pattern 21 | 0.75 | 0.01 | 0.724; [0.674 ... 0.762] |
| Fragmentation pattern 22 | 0 | 0.0005 | 0.625; [0.58 ... 0.663] |
| Fragmentation pattern 23 | 0.9 | 0.01 | 0.61; [0.535 ... 0.653] |
| Fragmentation pattern 24 | 1 | 0.01 | 0.662; [0.599 ... 0.703] |
| Fragmentation pattern 25 | 0.1 | 0.1 | 0.556; [0.518 ... 0.585] |
| Fragmentation pattern 26 | - | 0 | 0.587; [0.524 ... 0.624] |
| Fragmentation pattern 27 | 0.25 | 0.01 | 0.805; [0.75 ... 0.836] |
| Fragmentation pattern 28 | 0 | 0.1 | 0.603; [0.56 ... 0.653] |

**Table S8**. Selection of hyperparameters for Gaussian processes probabilistic classifiers.

| Variables | Hyperparameter | | | AUC value (median; [lower … upper quartile]) |
| --- | --- | --- | --- | --- |
|  | *θ_1_* | *θ_2_* | *Stdz** |  |
| The average fragment length | 5 | 1 | 1 | 0.685; [0.63 ... 0.734] |
| Standard deviation of the fragment length distribution | 10 | 0.1 | 0 | 0.608; [0.558 ... 0.665] |
| Standard error of the mean calculated for the fragment length distribution | 5 | 1 | 1 | 0.57; [0.512 ... 0.614] |
| Skew of the fragment length distribution | 50 | 1 | 0 | 0.62; [0.56 ... 0.659] |
| Kurtosis of the fragment length distribution | 5 | 0.1 | 0 | 0.637; [0.587 ... 0.669] |
| The derivative of the ratio “the number of short to the number of long fragments” | 50 | 50 | 1 | 0.638; [0.597 ... 0.671] |
| A number of short fragments | 1 | 0.5 | 1 | 0.699; [0.646 ... 0.738] |
| A number of long fragments | 50 | 5 | 1 | 0.687; [0.62 ... 0.718] |
| A total number of fragments | 10 | 50 | 1 | 0.678; [0.607 ... 0.721] |
| A number of short fragments corrected by GC-content | 5 | 50 | 1 | 0.704; [0.648 ... 0.751] |
| A number of long fragments corrected by GC-content | 50 | 10 | 1 | 0.667; [0.631 ... 0.713] |
| A total number of fragments corrected by GC-content | 10 | 10 | 1 | 0.668; [0.626 ... 0.707] |
| Fragmentation pattern 1 | 0.5 | 50 | 1 | 0.645; [0.587 ... 0.694] |
| Fragmentation pattern 2 | 1 | 5 | 1 | 0.653; [0.551 ... 0.701] |
| Fragmentation pattern 3 | 1 | 10 | 1 | 0.624; [0.579 ... 0.689] |
| Fragmentation pattern 4 | 0.1 | 5 | 0 | 0.577; [0.52 ... 0.636] |
| Fragmentation pattern 5 | 1 | 1 | 1 | 0.782; [0.738 ... 0.816] |
| Fragmentation pattern 13 | 1 | 0.1 | 1 | 0.686; [0.631 ... 0.73] |
| Fragmentation pattern 15 | 1 | 10 | 1 | 0.659; [0.621 ... 0.707] |
| Fragmentation pattern 16 | 1 | 10 | 1 | 0.645; [0.59 ... 0.692] |
| Fragmentation pattern 18 | 5 | 5 | 0 | 0.569; [0.514 ... 0.612] |
| Fragmentation pattern 20 | 5 | 0.1 | 1 | 0.761; [0.708 ... 0.796] |
| Fragmentation pattern 24 | 5 | 0.1 | 0 | 0.69; [0.635 ... 0.732] |
| Fragmentation pattern 27 | 1 | 0.5 | 1 | 0.788; [0.741 ... 0.837] |

**Stdz* value denotes centering and scaling of input data before the generation of the probabilistic classifier: “0” or “1” means that centering and scaling were not carried out or carried out, respectively.


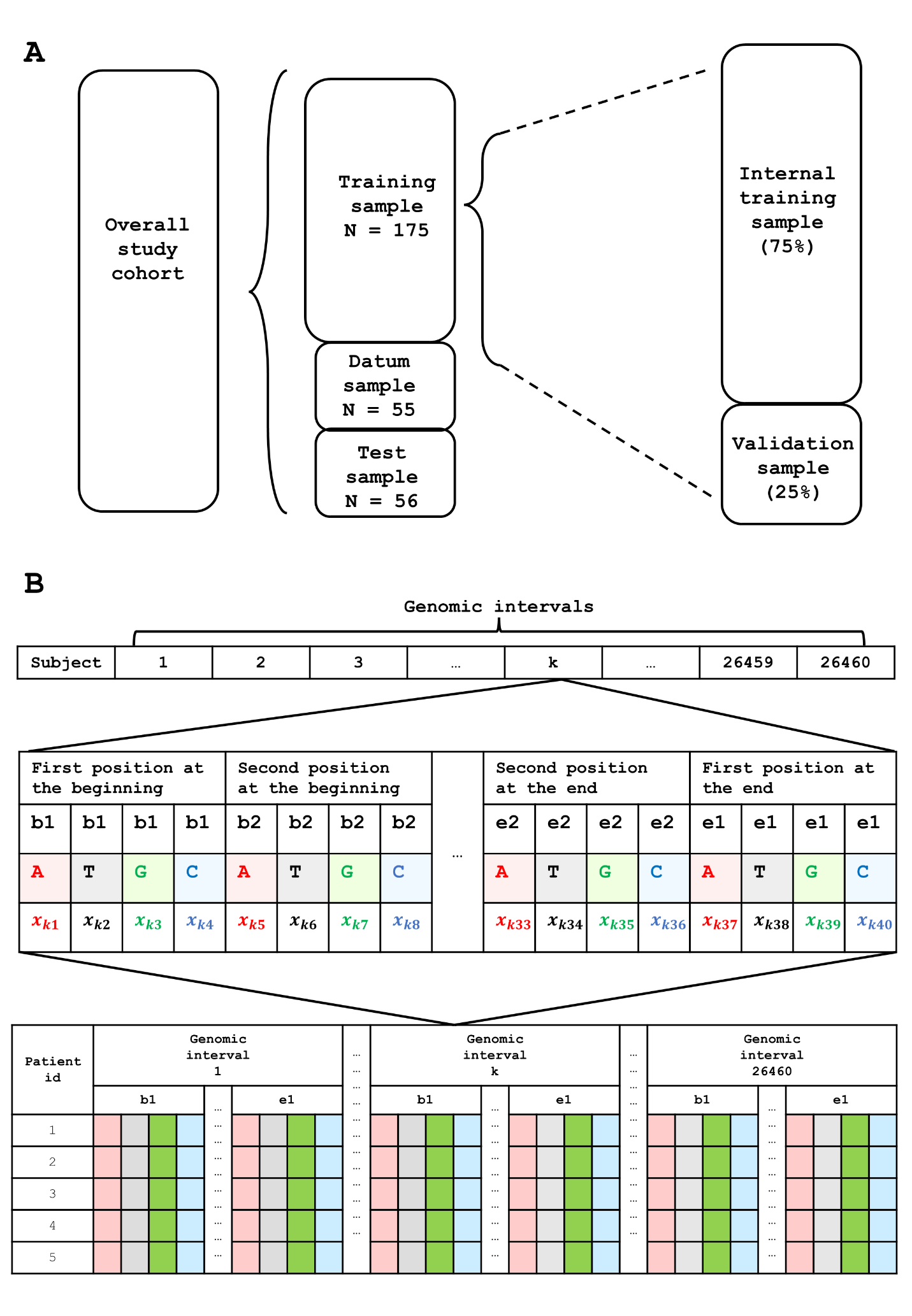


**Figure S1**. (A) – the partition of the original dataset into training, datum and test datasets. The partition of the training dataset into internal training and validation datasets during the hyperparameter optimization procedure. (B) – the partition of NGS-data into 26,460 non-overlapping intervals. This partition is the same for all subjects studied. Each section is characterized by 40 variables that correspond to the elements of the position-weight matrix (4 nucleotides * 2 fragment end motif * 5 positions at each fragment end motif).


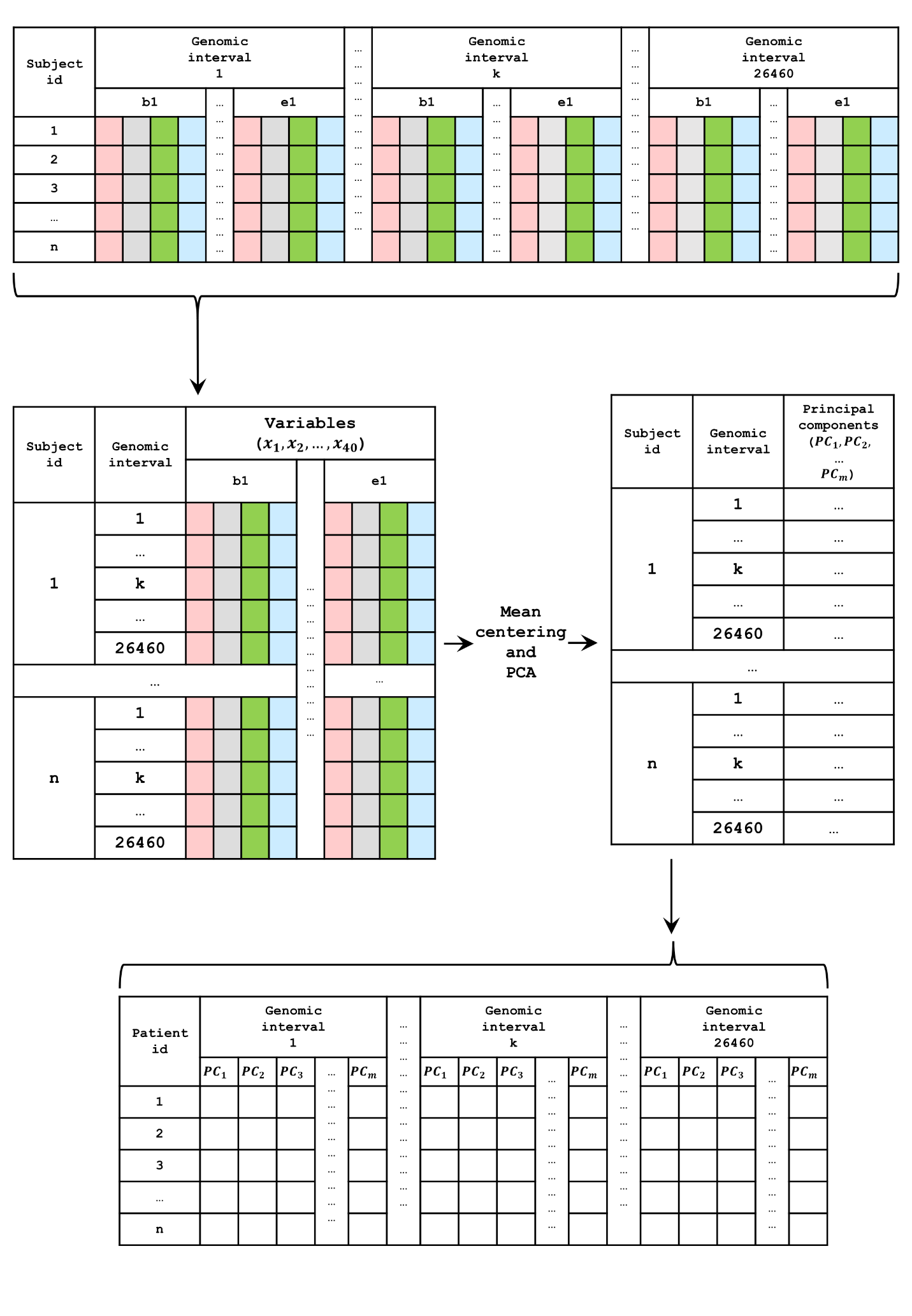


**Figure S2**. Reduction of dimension for data array containing characteristics of cfDNA fragments end motifs on a genome-wide scale using principal component analysis. The change in the dimension of the data array is shown before and after reduction of dimension. Designations: $x_{1}, x_{2}, \ldots, x_{40}$ – initial variables that are elements of the position-weight matrix, ${PC}_{1}, {PC}_{2}, \ldots, {PC}_{m}$ – calculated principal components.


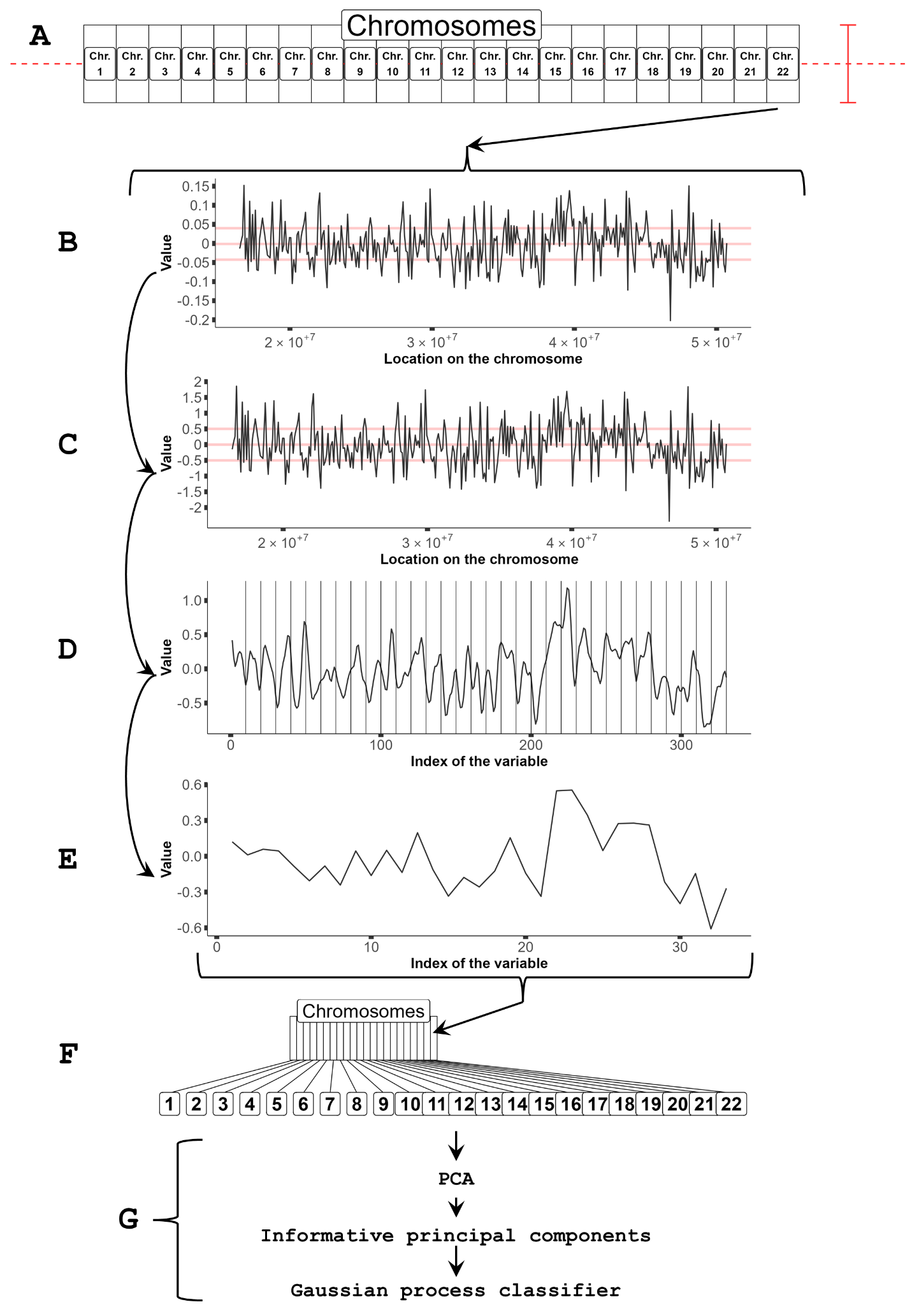


**Figure S3**. Reduction of dimension of input data for a Gaussian process classifier.

(A) – one characteristic corresponds to several thousand variables, for example, the number of short fragments or a fragmentation pattern. In turn, these variables correspond to genomic regions. Each chromosome covers several hundred variables.

(B, C) – centering and scaling performed within one observation. The median value of the characteristic is used as a measure of central tendency during the centering procedure. The interquartile range is used as a measure of dispersion in scaling. The median value and interquartile range of the characteristic are calculated for each subject on a genome-wide scale.

(D, E) – smoothing and averaging procedures.

(F) – transformed sets of values are combined into a new set of variables.

(G) – a new set of variables is transformed using principal component analysis. The selection of informative principal components is carried out. The resulting informative principal components are used as input variables for a probabilistic Gaussian process classifier.


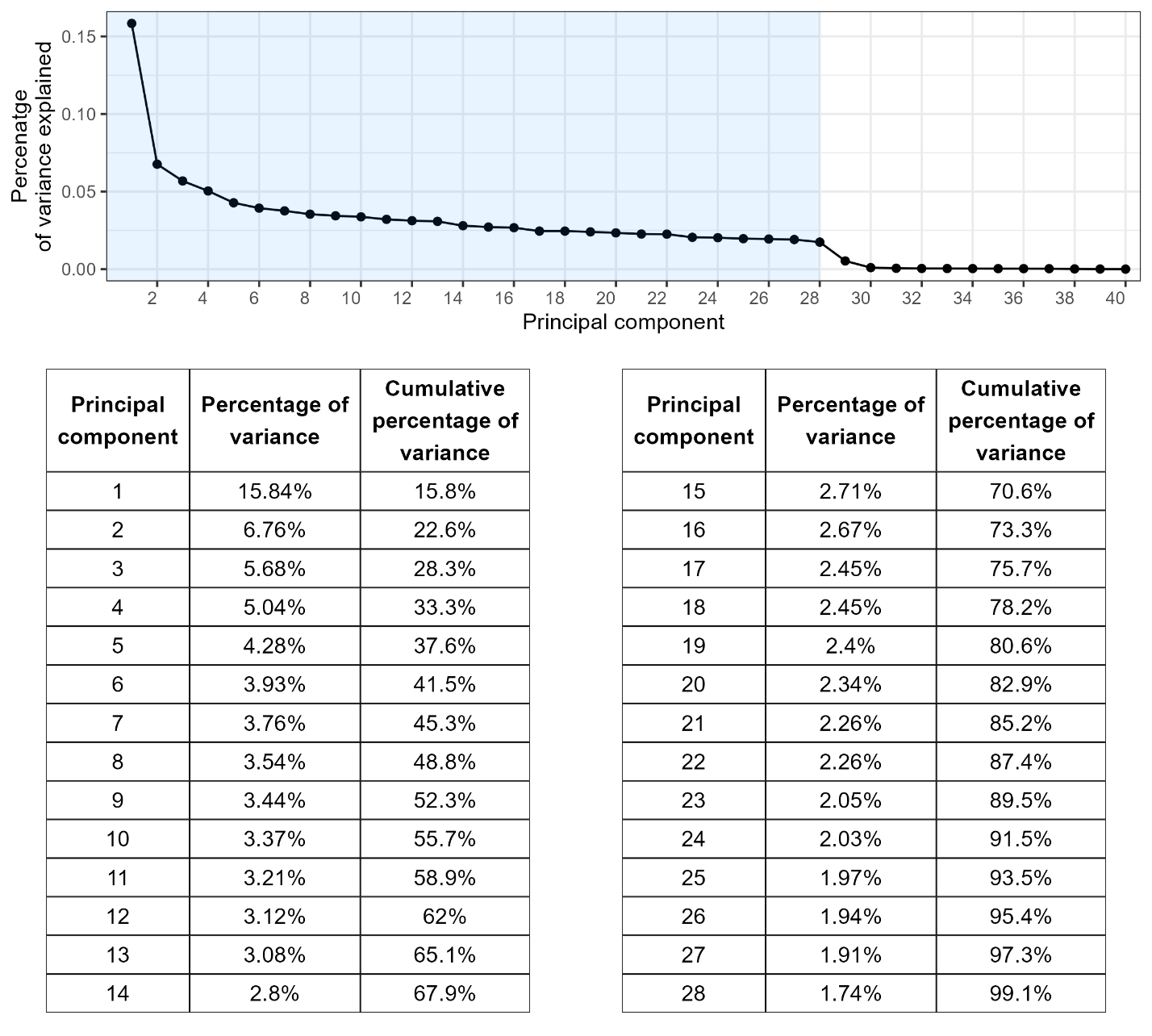


**Figure S4**. Results of calculation of fragmentation patterns. Based on the datum dataset, an array of 1,455,300 rows and 40 columns was generated and then transformed using principal component analysis. The resulting components are linear combinations of elements of position-weight matrix. A total of 28 informative principal components were selected, highlighted in blue color in the scree plot. The characteristics of informative principal components are presented in the table below the plot.
